# Robust and stretchable indium gallium zinc oxide-based electronic textiles formed by cilia-assisted transfer printing

**DOI:** 10.1038/ncomms11477

**Published:** 2016-06-01

**Authors:** Jongwon Yoon, Yunkyung Jeong, Heeje Kim, Seonggwang Yoo, Hoon Sun Jung, Yonghun Kim, Youngkyu Hwang, Yujun Hyun, Woong-Ki Hong, Byoung Hun Lee, Sung-Hoon Choa, Heung Cho Ko

**Affiliations:** 1 School of Materials Science and Engineering, Gwangju Institute of Science and Technology (GIST), 123 Cheomdangwagi-ro (Oryong-Dong), Buk-Gu, Gwangju 61005, Republic of Korea; 2 Graduate School of NID Fusion Technology, Seoul National University of Science and Technology, 232 Gongneung-ro, Nowon-Gu, Seoul 01811, Republic of Korea; 3 Jeonju Centre, Korea Basic Science Institute (KBSI), Jeonju, Jeollabuk-do 54907, Republic of Korea

## Abstract

Electronic textile (e-textile) allows for high-end wearable electronic devices that provide easy access for carrying, handling and using. However, the related technology does not seem to be mature because the woven fabric hampers not only the device fabrication process directly on the complex surface but also the transfer printing of ultrathin planar electronic devices. Here we report an indirect method that enables conformal wrapping of surface with arbitrary yet complex shapes. Artificial cilia are introduced in the periphery of electronic devices as adhesive elements. The cilia also play an important role in confining a small amount of glue and damping mechanical stress to maintain robust electronic performance under mechanical deformation. The example of electronic applications depicts the feasibility of cilia for ‘stick-&-play' systems, which provide electronic functions by transfer printing on unconventional complex surfaces.

## Abstract (web summary)

The fabrication of smart textiles is currently relying on the printing of ultrathin electronics on fabric, which is subject to the poor adhesion between electronics and textile. Here, Yoon *et al*. propose the use of cilia-like structure to improve adhesion in addition to release mechanical stress.

## Graphical abstract

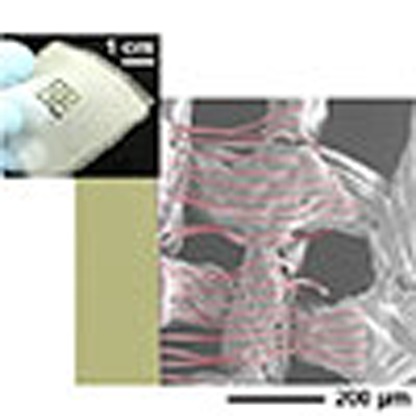

The recent technology of developing high-end flexible devices that are light^1^, imperceptible^2^, deformable^3^,^4^, wearable^5^,^6^,^7^,^8^,^9^,^10^,^11^,^12^,^13^,^14^,^15^,^16^,^17^,^18^, attachable^5^,^6^,^7^,^19^ and/or disposable^20^ offers users a high degree of freedom in keeping, carrying, using and discarding devices. Electronic textile (e-textile) has the unique advantages of using a woven texture that fits the human body comfortably, deforming naturally upon movement, and being permeable to air and sweat^21^,^22^. To develop an e-textile, two kinds of approaches are possible. One is to create one-dimensional thread-like electronic devices and interlace them, as in the examples of energy saving/converting systems^8^,^9^,^10^, light-emitting devices^11^,^12^, simple woven logic^23^ and sensors^8^,^13^,^24^, which do not require high-density pixel arrays or highly integrated circuits. The other is to fabricate a two-dimensional ultrathin device, such as an antenna^25^, a logic circuit^26^, an electrochromic device^14^, tactile/epidermal sensors^15^,^27^ or an energy-harvesting device^16^,^17^, and print it onto a premade textile. This strategy allows for a wide window of device fabrication including conventional wafer-based process technology that uses, for example, photolithography, etching, deposition and other techniques to fabricate high-performance electronic devices^28^.

Judging from the architecture of electronic devices transferred onto complex substrates, including textile^6^,^14^,^15^,^16^,^17^,^26^,^27^, human skin^29^,^30^,^31^,^32^, animal organs^31^,^32^ or brains^33^,^34^, a leaf, a contact lens^1^,^35^ and a hemispherical substrate^36^,^37^, substantial effort has been devoted to accommodating the interface mismatch. First, as rule of thumb, a polymer substrate (for example, silicon rubber^5^,^6^,^24^,^26^,^32^,^33^, SU-8 (refs 19, 27), parylene^1^,^18^ or polyester^29^) should be as thin as possible for efficient conformal contact on the target surfaces^34^. Second, the device should be made robust to repeated large deformations by using, for example, elastic materials^3^,^5^, dispersion of conducting nanowire/platelet type^33^,^35^ and/or compressible/stretchable buckled/serpentine electrical interconnects^38^,^39^. Third, the adhesion should be sufficiently strong, but caution should be used to conserve the characteristic features of the substrates. For example, using too much glue or full coverage of an additional layer on a textile can promote interfacial adhesion but can easily lose the advantages of the textile^14^,^15^,^16^,^26^.

This study, in particular, addresses the third issue. We envision that the introduction of a lateral cilia structure in the periphery of a substrate, which can reliably wrap the nearby threads of a textile, would allow for the conformal transfer of a device on a textile. Moreover, the cilia confine glue near themselves rather than in the cilia-free region and can markedly promote interfacial adhesion with an extremely small amount of glue. To confirm the conceptual features of this method, we examined the enhancement of interfacial adhesion using cilia with diverse length scales and densities, together with glue, and successfully transferred diverse substrate patterns (triangle, square, circle and alphabets) with diverse sizes (ranging from 300 × 300 μm to 1 × 1 cm) onto a bandage, a metal tea ball strainer, a cotton swab and a stone. Furthermore, reliability testing of the performance of electronic devices upon the deformation of various e-textiles, together with mechanical analysis, reveals that cilia serve as dampers to release stress during mechanical deformation. Using this technology, we successfully developed textile-type indium gallium zinc oxide (IGZO)-based transistors, inverters and a ring oscillator that are robust to harsh environmental conditions such as mechanical stretching, wearing in daily life, and washing with detergent and water.

## Results

### Cilia-assisted transfer printing process

**Figure 1:**
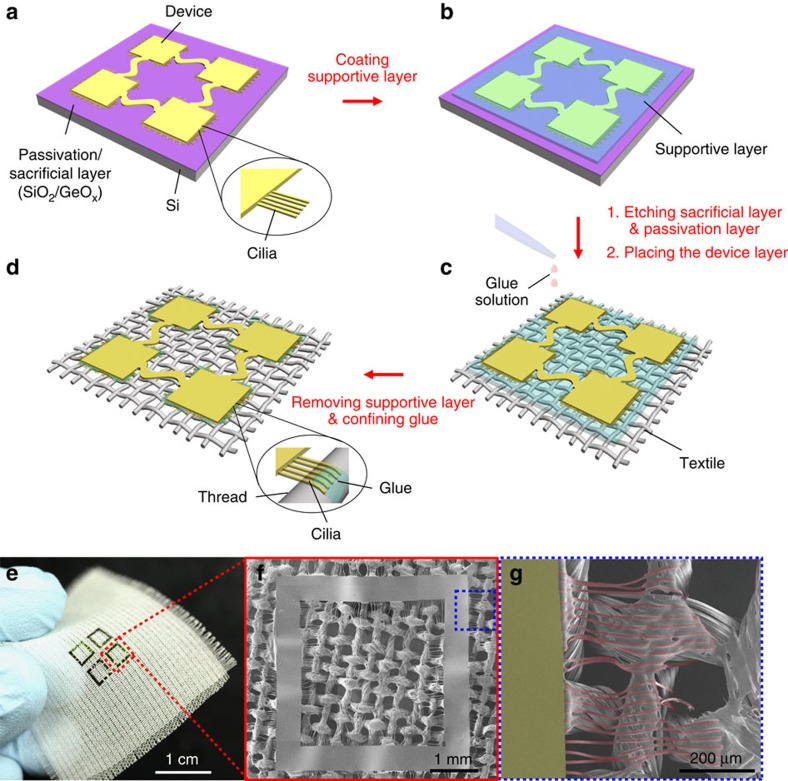
Schematic procedure for cilia-assisted transfer printing. (**a**–**d**) Illustration of steps for transfer printing of ultrathin devices with peripheral cilia onto a textile. (**e**–**g**) Photograph (**e**) and selected SEM (**f,g**) images of 2 × 2 open square pattern arrays of PI (thickness=2 μm)/Cr (70 nm)/PI (2 μm) layered structure with PI (thickness=1.8 μm) peripheral cilia transferred onto a textile. The image (**g**) was colorized with olive (corresponding to main substrate) and red (corresponding to peripheral cilia) to enhance the contrast between variable regions.

Figure 1 shows the transfer printing process of a stretchable ultrathin device from a handling substrate to a textile using peripheral cilia. The process begins with coating a GeO_*x*_ sacrificial layer and a SiO_2_ protective layer on a handling substrate; a Si wafer is used in this study. After coating the entire area of the substrate with polyimide (PI, Sigma-Aldrich, thickness=2 μm), it is possible to fabricate an electronic device using conventional methods (see the detailed device fabrication steps in Supplementary Fig. 1). After device fabrication, an additional PI (thickness=2 μm) layer was added to protect the device; the thickness of the top PI layer was the same as the bottom PI layer to provide the device layer with a neutral mechanical plane^40^. After reducing the thickness of the cilia region to the desired value by reactive ion etching (RIE, O_2_, 20 s.c.c.m., 50 mtorr, 150 W), the PI layers, except for the area corresponding to the device and the cilia, were etched away (see Fig. 1a and Supplementary Fig. 2). Spin coating with a solution of poly(methyl methacrylate) (PMMA, MicroChem, *M*_W_=950,000 g mol^−1^) creates a supportive layer to sustain the overall shape of the ultrathin device for the remaining transfer printing process (Fig. 1b). After etching the sacrificial layer in water at 70 °C, the device was peeled from the handling substrate and placed onto a textile for further attachment processing; the PMMA layer faces the textile unless mentioned otherwise (Fig. 1c). To promote adhesion in the initial stage, the textile was pre-wetted with additional polydimethylsiloxane (PDMS) precursor solution (curing agent=10:1 by weight) diluted with toluene. Repeatedly dropping the PDMS solution on top of the device gradually dissolves and washes away the PMMA layer; the cilia maintain a planar structure in the early stage and start to bend when this process completely dissolves the supportive glue framework and washes away most of them (Supplementary Fig. 3). After removing the PMMA layer completely, the residual PDMS glue solution was first dried at room temperature and then annealed on a hotplate at 120 °C for 2 h to form the final e-textile (Fig. 1d). During the dissolving and drying process, the cilia gradually wrap onto nearby threads by capillary condensation^41^; the surface tension of the liquid induces a meniscus and viscous forces and maintains the interfacial adhesion between the cilia and threads. After complete drying of the glue solution, the cilia confine the glue nearby rather than on the main substrate. The photograph and scanning electron microscope (SEM) images in Fig. 1e–g demonstrate that the combination of the wrapping behaviour of cilia near threads and the confinement of glue around the cilia markedly enhances the selective adhesion to enable the overall transfer printing of planar devices on woven surfaces.

### Enhanced adhesion of substrates by cilia and glue

**Figure 2:**
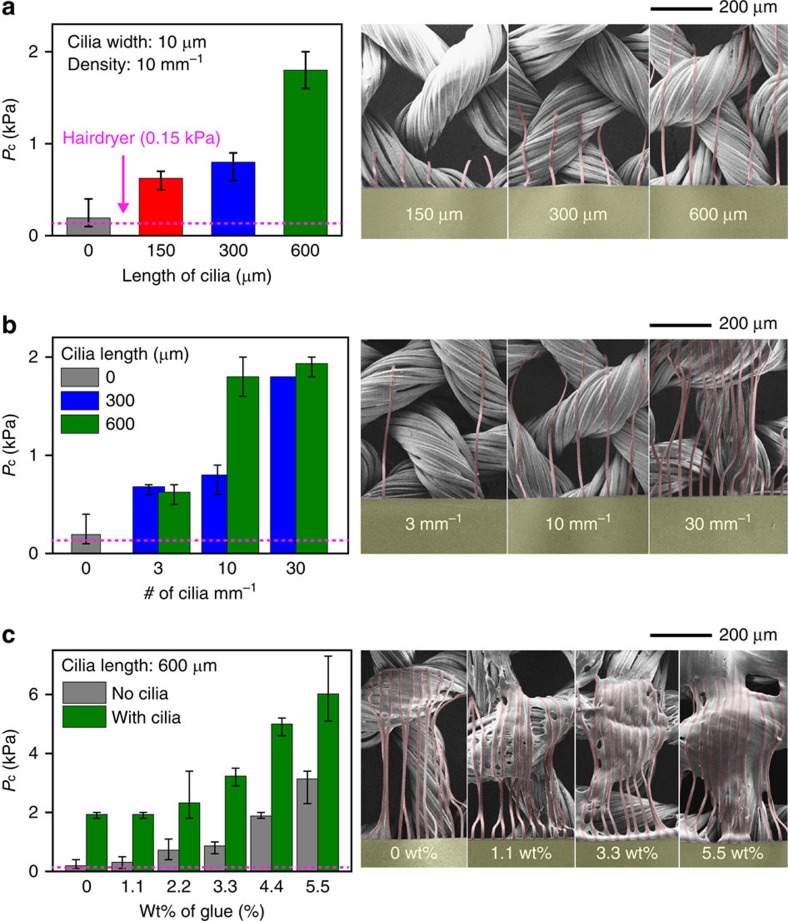
Enhanced adhesion beneath ultrathin substrates by cilia and glue. (**a**–**c**) Blowing test for measuring critical pressure (*P*_c_) of the substrates with various cilia length scales (**a**), cilia densities (**b**) and concentrations of PDMS glue solution in toluene (**c**) for different six samples. The thickness and width of the cilia were set to 1.8 and 10 μm, respectively. The olive and red regions represent PI (thickness=2 μm)/Cr (70 nm)/PI (2 μm) and PI (1.8 μm), respectively. The Cr interlayer was added for improving the optical contrast. The dashed lines at 0.15 kPa, corresponding to the most intensive air blowing achievable using a conventional hairdryer, were added for comparison.

To determine the contribution of the cilia to the interfacial adhesion between a transferred substrate and a target surface, a detachment test was conducted by blowing air on the 2 × 2 PI open square pattern arrays shown in Fig. 1e with various cilia length scales and density; the substrates began to be detached at the critical pressure (*P*_c_); see Fig. 2, Supplementary Fig. 4, Supplementary Note 1 and Supplementary Movie. To investigate the contribution of the cilia to *P*_c_ sufficiently, the length and density were varied from 0 to 600 μm (fixed width=10 μm, fixed density=10 cilia per mm) and from 0 to 30 cilia mm^−1^ (fixed length=600 μm, fixed width=10 μm), respectively. The thickness of the cilia was adjusted to 1.8 μm in this study to guarantee conformal wrapping and mechanical strength during the transfer process because thick cilia (for example, thickness >3 μm) are too stiff for conformal wrapping, while thin cilia (thickness <1 μm) are not robust against mechanical stress (see the SEM, optical microscope images and peel test in Supplementary Figs 5–7 and Supplementary Note 2). In the beginning, pure toluene was used to remove the supportive layer completely in step Fig. 1c to examine the contribution of cilia alone. *P*_c_ gradually increases with the length and density of cilia; see the values (0.2, 0.6, 0.8 and 1.8 kPa) for varied length scales (0, 150, 300 and 600 μm) in Fig. 2a and the values (0.2, 0.6, 1.8 and 1.9 kPa) for varied numbers of cilia per mm (0, 3, 10 and 30) with a fixed cilia length of 600 μm in Fig. 2b. *P*_c_ increased slightly by 11% (that is, 1.7–1.9 kPa) when the sample with cilia (fixed length=600 μm, fixed width=10 μm and fixed density=30 mm^−1^) was flipped over for the transfer process (Supplementary Fig. 8; Supplementary Table 1). Most of the cilia with no glue were unwrapped from the threads upon intensive air blowing (Supplementary Fig. 9; Supplementary Note 3). To enhance the adhesion between the cilia and textile, we used an additional glue solution instead of toluene in step Fig. 1c. The higher the concentration of glue precursor solution used, the more residual glue remains near the cilia in the final state (Supplementary Fig. 10), increasing *P*_c_; see the values (1.9, 2.0, 2.6, 3.2, 5.0 and 6.0 kPa) for varied concentrations of PDMS precursor solution (0, 1.1, 2.2, 3.3, 4.4 and 5.5 wt%) with fixed length, width and density of cilia (600 μm, 10 μm and 30 mm^−1^, respectively; Fig. 2c); see more detailed analysis and discussion in Supplementary Figs 11-16 and Supplementary Notes 4–7. Furthermore, most of the cilia prepared using PDMS precursor solutions of not less than 3.3 wt% were torn rather than unwrapped upon intensive air blowing, confirming that the additional glue enhances the adhesion between cilia and threads strongly enough for further study (Supplementary Fig. 9). The non-uniform distribution of residual glue, highly confined near the cilia but almost absent at the top and bottom of the main substrate, also supports the role of cilia in transfer printing on textile (see the photograph and SEM images in Supplementary Fig. 17).

### Mechanical analyses for enhanced adhesion by cilia and glue

**Figure 3:**
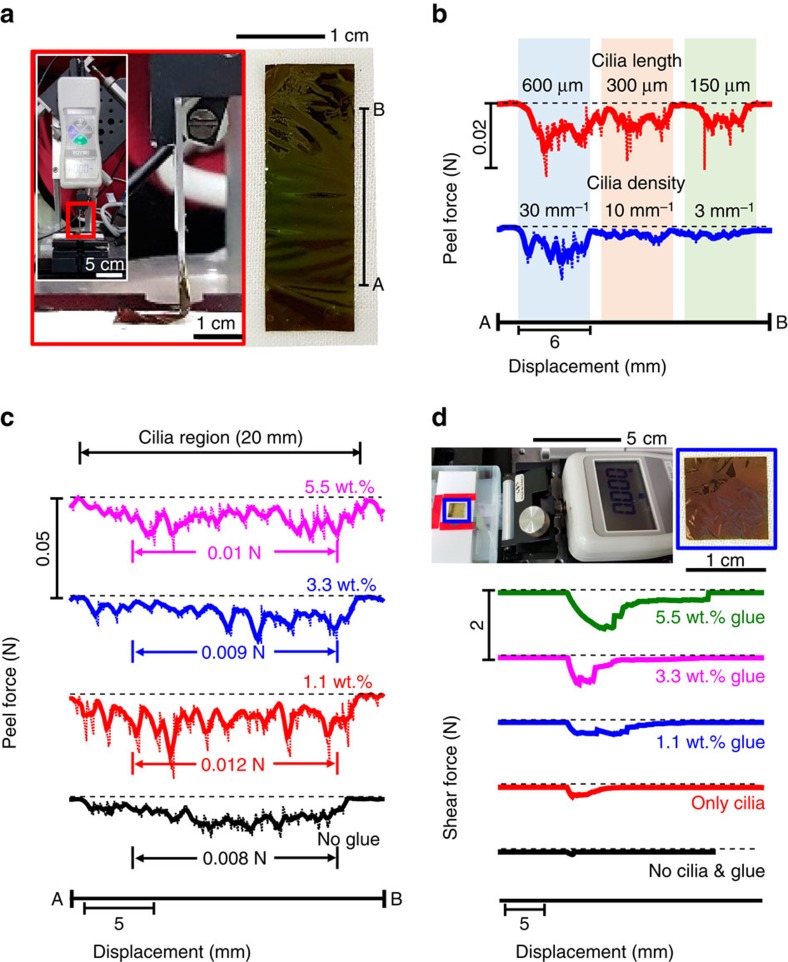
Mechanical analyses of the enhanced adhesion by cilia and glue. (**a**) Photographs of the measurement set-up (**a**-left) and a substrate (**a**-right) used in the peel test. (**b**,**c**) Peel force profiles of the substrates with various cilia length scales (**b**-top), densities (**b**-bottom) and concentrations of the PDMS glue solution in toluene (**c**). (**d**) Photographs of the measurement set-up and a substrate used in the shear test (**d**-top) and the shear force profiles of the substrates with or without cilia (thickness=1.4 μm, width=10 μm, length=600 μm, density=30 mm^−1^) and various concentrations of PDMS glue solution in toluene (**d**-bottom). Shear rate is set to be 0.3 mm s^−1^. The textile used in these mechanical tests is the same as that used in Fig. 2.

Peel and shear tests of the substrates with/without cilia and glue provide profound insight into the role of the cilia in the interfacial adhesion. Figure 3a shows photographs of the measurement set-up (left) and a representative PI substrate (1 × 3 cm, right), including cilia regions on the two longer sides. The peeling rate and angle were set to 0.3 mm s^−1^ and 90°, respectively. For the case of using only cilia without glue, the peel force increases with the cilia length and density (Fig. 3b), which is the same tendency as shown in Fig. 2a,b. We believe the longer and denser cilia naturally provide a higher probability of enlarging the contact area between the cilia and textile, resulting in stronger adhesion. The peel force slightly increases as a result of the use of glue but has similar values for the various concentrations of the glue solution; see the average peel forces of ∼0.01 N. Furthermore, when the concentration of the glue solution is fixed at 3.3 wt%, the peel force increases significantly with the thickness of the cilia (Supplementary Fig. 18). We believe that this behaviour is because the use of the dilute glue solution induces partial or inhomogeneous coverage of the glue around the cilia, and the peeling of the substrate thereby occurs by the delamination or fracture of the cilia in sequence at the glue-free/deficient region in the cilia.

To investigate a different mode of damaging the e-textile, we conducted shear tests by moving the substrates (main substrate area=1 × 1 cm, cilia (fixed length=600 μm), fixed width=10 μm and fixed density=30 mm^−1^) introduced on all sides of the main substrate) at a shear rate of 0.3 mm s^−1^. Compared with the finding in the peel test, the shear force increases much more significantly with the concentration of the glue solution. There are two factors of concern. First, this shear mode leads to the delamination or fracture of the entire cilia in a synchronized manner much more than the peel mode does. Second, the four directional alignments of the peripheral cilia contribute to the shear force in a complex manner. We believe the promoted adhesion of the cilia to the nearby threads enriches the overall tension of the entire cilia in such a synchronized manner and provides more stability in complex deformation. The results of the peel and shear tests indicate the utility of the e-textile in our method; for example, the embedding of an e-textile inside the sleeve, the collar or the placket of a shirt will take advantage of the use of cilia and glue.

### Examples of cilia-assisted transfer printing on complex surfaces

**Figure 4:**
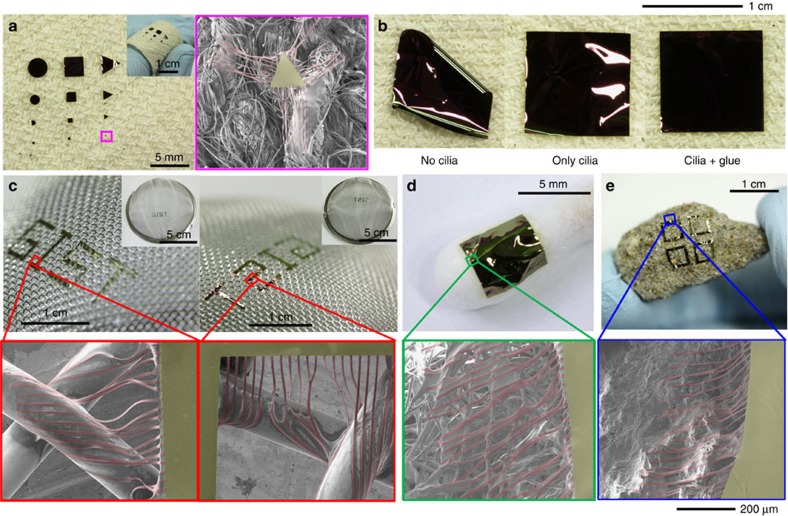
Examples of transfer printing on complex surfaces. (**a**–**e**) Photographs and/or magnified SEM images of various PI patterns with cilia (thickness=1.8 μm, width=10 μm, length=600 μm, density=30 mm^−1^) transferred onto a bandage (**a**,**b**), a tea strainer (**c**), a cotton swab (**d**) and a stone (**e**). Diluted PDMS solution (3.3 wt% in toluene) was used as glue. During the transfer process, the PMMA layer faces the target substrates except for the sample used in **c**-right. The PI patterns other than cilia were deposited with a Cr layer (thickness=70 nm) for high optical contrast.

The method of cilia-assisted transfer printing is accessible for diverse sizes and patterns of PI substrates. For example, circles (diameter=300 μm−2.5 mm), squares (side=300 μm−2.5 mm) and triangles (side=300 μm−2.5 mm) can be successfully transferred onto a bandage with no significant distortion (Fig. 4a), whereas PI patterns with no cilia become easily detached and roll up from the surface (Supplementary Fig. 19). Conformal printing at the centimetre scale is possible (Fig. 4b); the combination of cilia and glue guarantees strong adhesion around the cilia and thereby no delamination at the periphery, whereas the no-cilia and cilia-only devices exhibit a rolled-up shape and slight delamination, respectively. Other complex surfaces are also feasible. For example, we successfully transferred a PI substrate with the letters ‘GIST' onto a tea strainer regardless of the facing side of the PI substrate towards the strainer (Fig. 4c). More examples of conformal printing onto a cotton swab and a stone with random and complex morphology show the versatility of cilia and glue for the transfer printing of ultrathin devices onto complex nonlinear surfaces (Fig. 4d,e; Supplementary Fig. 20).

### Enhanced durability of stretchable electrodes by cilia

**Figure 5:**
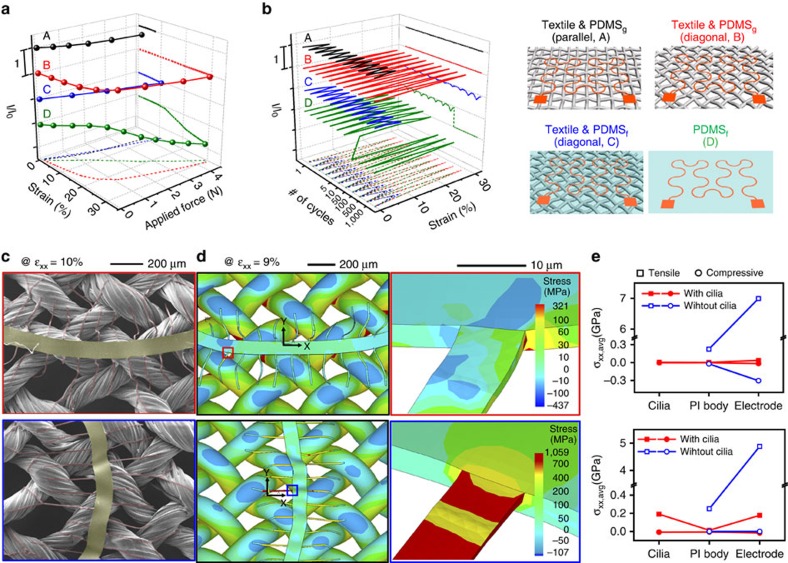
Enhanced durability of electrodes upon stretching by cilia. (**a**,**b**) Strain–stress *I*/*I*_0_ characteristics upon one-dimensional stretching (**a**) and electrical fatigue test upon repeated stretching up to∼3 (± 0.5) N(corresponding strains for samples A to D=6.8%, 28.5%, 8.5% and 25.6%, respectively) and releasing for 1,000 cycles (**b**) for four different samples. All encapsulated electrodes used in this test consist of PI (2 μm)/Cr (5 nm)/Au (70 nm)/PI (2 μm) with peripheral cilia (PI (1.8 μm)). The electrodes transferred onto textiles with two types of alignment (noted by parallel (A) and diagonal (B and C)) using 3 wt% PDMS glue solution in toluene. Sample C was prepared by an additional coating of PDMS film (total thickness=400 μm). Sample D was prepared by transfer printing on PDMS film followed by additional coverage of PDMS film (total thickness=500 μm). (**c**) SEM images of sample B in selected area placed on the textile parallel (top) and perpendicular (bottom) to the stretching direction (*x* axis) at tensile strain *ɛ*_xx_=10%. (**d**) Stress distribution maps for the sample B obtained by FEM simulation at tensile strain *ɛ*_xx_=9%; the red and blue boxes correspond to the magnified stress distribution maps of the selected areas. (**e**) Average stress (*σ*_xx,avg_) of each component of the sample B and the electrode without cilia, placed on the textile parallel (top) and perpendicular (bottom) to the stretching direction (*x* axis), at tensile strain *ɛ*_xx_=9%; for the case without cilia, the adhesion between the textile and main substrate is set to ideal bonding state.

The combined mechanical behaviour of cilia and textile provides a profound insight into the development of robust e-textiles. To allow systematic analysis, we used a universal form of a printable serpentine metal electrode consisting of PI (2 μm)/Cr (5 nm)/Au (70 nm)/PI (2 μm) with peripheral cilia and 3 wt% of PDMS glue solution in toluene or acetone; it is expected that the synergetic motions of serpentine connection lines and the peripheral cilia promote stretchability with high stability. Based on the electrode, four types of stretchable electrodes were prepared considering the target substrates (textile or PDMS film), existence of additional coverage of PDMS film (PDMS_g_ for residual PDMS glue only and PDMS_f_ for additional PDMS film) after transfer printing, and alignment (‘parallel' or ‘diagonal') of the electrode compared to the weft thread (top right in Fig. 5); see the illustrations for samples A, B, C and D corresponding to textile and PDMS_g_ (parallel), textile and PDMS_g_ (diagonal), textile and PDMS_f_ (diagonal) and PDMS_f_, respectively. Figure 5a shows the relative current ratio (*I*/*I*_0_)-strain-applied force curves of the four types of electrodes, where *I*_0_ and *I* are current values of the electrodes before and after stretching the samples, respectively; see the magnified optical microscope images of the electrodes stretched up to a tensile force of 4 N in *x* axis direction (Supplementary Fig. 21). As expected of the mechanical behaviour of a textile that naturally allows slight but not excessive deformation, all three types (A and B) using textile substrates show remarkable nonlinear strain-applied force curves. The ratio of applied force to strain can be varied depending on the textile and stretching direction; for example, sample A can be stretched up to 7.5% (*I*/*I*_0_=3.1%) at a tensile stress of 3.8 N, whereas sample B can be stretched much more, up to 30.4% (*I*/*I*_0_=4.7%), at a tensile stress of 4.4 N. Sample C shows similar mechanical behaviour to sample A due to the combined mechanical effect of a textile and additional PDMS_f_, and the value of *I*/*I*_0_ decreases more significantly, by 6.2%, even though the strain is only 10.6% at a tensile force of 4.5 N. Sample D, with no textile substrate, shows an 87.3% decrease in *I*/*I*_0_ at a strain of 29.4% (tensile stress=4.4 N). To clarify the stability of the electronic performance of the four samples, we performed a fatigue test of the four samples by stretching up to 3 N (±0.5 N) and releasing in a dynamic mode for 1,000 cycles (Fig. 5b; Supplementary Fig. 22). Samples A and B showed slight variations in *I*/*I*_0_ within 1.8 and 1.2%, while samples C and D showed a significant decrease of *I*/*I*_0_ by 26.2% and even electrically disconnection, respectively. We also performed a fatigue test for an additional 1,000 cycles after intentionally generating a cut by scissors at the edge of textile used for each sample. Samples A and B still maintain their *I*/*I*_0_ values with a slight decrease of 3% for both, whereas sample C shows electrical disconnection at the 494th cycle, even though there was no crack propagation in the textile substrate. For the new sample D, electrical failure occurred after only 3rd cycles.

To investigate the damping effect of cilia during the mechanical deformation, we performed the numerical stress and strain analysis using the finite elemental method (FEM). Numerical mechanical modelling and material properties used in the study are described in Supplementary Fig. 23, Supplementary Table 2 and Supplementary Note 8. We representatively consider electrodes corresponding to sample B in Fig. 5a,b. Upon 10% tensile strain parallel to main substrate, the curved electrode becomes less wavy whereas the cilia are more wrinkled, and vice versa for stretching mode in perpendicular direction (Fig. 5c). FEM modelling was constructed on the base of the SEM images to figure out the stress distribution of each component after stretching of 9% tensile strain (Fig. 5d; Supplementary Fig. 24). The stress-releasing effect of the peripheral cilia is clearly illustrated on Fig. 5e. Compared with the case without cilia at ideal bonding state between the textile and main substrate, the average stress (*σ*_xx,avg_) of electrode is reduced from 7 GPa (without cilia) to 37 MPa (with cilia) in parallel position and 5 GPa to 177 MPa in perpendicular position; the failure stress of Au is ∼300 MPa (ref. 42). The maximum stress (*σ*_xx,max_) occurs mainly on the connected area between the PI substrate and peripheral cilia as shown in Fig. 5d. Some of the cilia show the tensile *σ*_xx,max_ of several hundred MPa, which is much larger than the failure stress (77 MPa) of PI (Supplementary Fig. 25)^42^; see the magnified stress distribution map highlighted in Fig. 5d bottom. When some of the cilia scarify to release accumulated stress (Supplementary Fig. 26), the remaining cilia will redistribute the stress of the electrode, which results in the improvement of stability and robustness of the electronic devices attached on the textile.

### Mass and thermal transfer characteristics through e-textile

The partial coverage of the elastomer glue near the cilia in sample PDMS_g_ provides ease of mass transfer through the e-textile. For example, the permeation of water vapour through PDMS_g_ (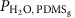) reaches 83% compared with the value of the textile only; 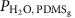
=1.9 mg cm^−2^ s^−1^, 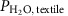
=2.3 mg cm^−2^ s^−1^ and 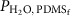
=0.053 mg cm^−2^ s^−1^, as shown in Supplementary Fig. 27 and Supplementary Table 3. Regarding ability of heat dissipation, use of small amount of the elastic glue in sample PDMS_g_ allows very shorter decay time, *t*, required to reach 80% at maximum temperature (48–51 °C), compared to sample PDMS_f_; the values were 13.0 and 23.9 s, respectively, under natural air-cooling conditions with no fan (see more detailed analysis in Supplementary Fig. 28, Supplementary Tables 4–5 and Supplementary Note 9).

### 7-Stage ring oscillator on textiles

**Figure 6:**
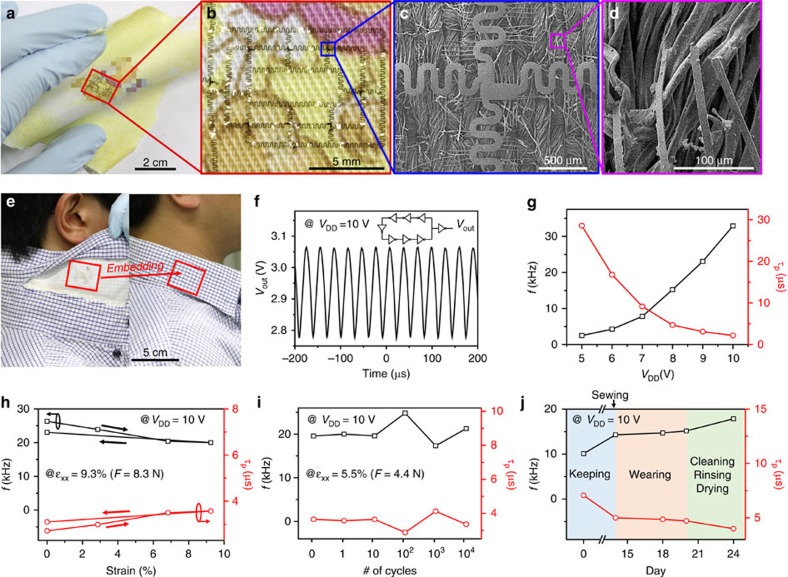
IGZO-based 7-stage ring oscillator transferred onto textiles. (**a**–**d**) Photograph and magnified SEM images of the device transferred on a handkerchief. (**e**) Photographs of the device transferred onto a piece of cloth and sewn inside the collar of a shirt. (**f**,**g**) Representative output voltage waveform at *V*_DD_=10 V (**f**) and oscillation frequency and propagation delay versus different *V*_DD_ values (**g**) of the device. (**h**–**j**) Oscillation frequency and propagation delay upon tensile deformation (**h**), after repeated stretching (up to∼4.4 N) and releasing (**i**), and after wearing and cleaning/rinsing/drying (**j**).

As an example of an e-textile using the new cilia-assisted transfer printing technology, we prepared an ultrathin IGZO-based 7-stage ring oscillator (RO) with peripheral cilia (length=600 μm, width=10 μm, density=20–25 cilia per mm). For circuit design, the device has inverters, each of which consists of an enhancement-type drive TFT (*L*=10 μm, *W*=120 μm) and a load TFT (*L*=10 μm, *W*=30 μm) with a beta ratio [*β*=(*W*_drive_/*L*)/(*W*_load_/*L*)] of 4; see the photograph image and equivalent circuit diagram of RO, and the basic electrical properties of IGZO-based TFTs and inverters in Supplementary Figs 29–30 and Supplementary Note 10. The device was transferred onto a handkerchief using 3 wt% PDMS glue solution in toluene (Fig. 6a,b) as a representative mobile e-textile. As expected, the cilia anchor to the threads underneath and maintain the lateral position of the main electronic device, as shown in the SEM images in Fig. 6c,d. e-Textiles can be applied for wearable electronic devices, as shown in the example sewn into a shirt collar (Fig. 6e). The textile used here has a denser woven structure than the sample in Fig. 5b; under a tensile stress of 4.4 N, the available strain level becomes 5.5%, which is much lower than in Fig. 5b (30.4%). At *V*_DD_=10 V, output voltage oscillates with a frequency (*f*) of 33 kHz and a propagation delay (*τ*) of 2.2 μs, as shown in Fig. 6f; *τ* is calculated by 1/(2·*n*·*f*), where *n* is the number of stages, and *f* increases with *V*_DD_ (5 to 10 V, step=1 V, Fig. 6g)^43^. We expect to increase *f* by reducing the parasitic capacitance and resistance, such as minimizing the overlap distance between the gate and source/drain electrodes and enlarging the cross-sectional area while shortening the contour length of the serpentine interconnect lines. To confirm the robustness of the 7-stage RO upon mechanical deformation in a tensile mode, we monitored *f* at *V*_DD_=10 V under various tensile stresses up to 8.3 N (tensile strain=9.3%, Fig. 6h) (see Supplementary Fig. 31 for the device transferred onto a textile in the parallel direction), and 10,000 repeated cycles of tensile stress ranging from 0 to 4.4 N (tensile strain=5.5%, Fig. 6i). Upon stretching, *f* decreased with tensile strain and did not fully recover to the initial value after 1 cycle of stretching. We believe that such mechanical deformation could create micro/nano-scale defect domains, particularly in the regions near the main electronic device, nonlinear serpentine interconnect lines and external contact pads with silver paste, thereby cause an IR drop and reducing the actual input voltage; we also observed that additional silver paste coating often reduces the variation in *f* value. When applying repeated tensile strain between 0 and 5.5% (corresponding to a tensile stress of 4.4 N) to the device for up to 10,000 cycles, there were no significant changes in *f*. To confirm the mechanical durability of the device in Fig. 6d for use in real daily life, one of the authors used the shirt personally throughout each day for 1 week while monitoring the electronic function (Fig. 6j). The device worked without significant retardation in *f*. Furthermore, the durability in wet conditions for laundry (detergent diluted with water) also demonstrates the potential of our technology for wearable e-textiles; it should be noted that the device survives during simple dipping but cannot survive significant crack formation together with the failure of electronic function during laundering in a washing machine.

## Discussion

The ability of cilia to wrap curvilinear structures and confine glue nearby allows random access to planar ultrathin electronic devices for complex geometries with paradoxical surface mismatch. For successful cilia-assisted transfer printing, it is desirable to select appropriate materials for a sacrificial layer, an etchant to remove the sacrificial layer, a support layer and a solvent to dilute the rubber precursor solution. For example, none of the chemicals should damage any parts of the created device or the target surfaces. The etchant and the dilution solvent should selectively remove the sacrificial layer and the support layer. The use of a proper fabrication procedure is also important. After sufficient dropping of the glue solution, a further annealing process should guarantee both the removal of the solvent for dilution and the complete curing of the rubber precursor. Because the vaporization rate of the solvent and the curing rate generally increase with the annealing temperature, it is necessary to prolong the annealing process at a lower temperature to ensure a mild thermal history. The use of the dilute PDMS glue to promote cilia adhesion may block the fabric pores, particularly when the fabric density becomes higher. We believe that dropping the glue solution in a specific area by advanced technology such as ink-jet printing is also an alternative option to minimize the coverage of the glue.

The damping behaviour of the cilia under mechanical deformation enhances the robustness for the use of e-textiles in daily life. Introducing a cilia structure with desired parameters such as the length, density, shape and thickness, and designing an appropriate pattern for the main substrate are important, because too large a main substrate can suffer from delamination, and too many cilia limit the available area for device integration. We believe that the application example shows the feasibility of our method for the development of e-textiles and various curvilinear electronic applications. To develop advanced e-textiles with, for example, an additional power supply, control units, wireless data transmission parts and memory modules, the e-textile has to be connected with outside bulk electronics, and the contact junctions and wiring have to be protected to prevent electrical leakage and mechanical damage during daily activities, including the washing of the e-textile. We believe that the further development of plastic or elastic conductors with a low Young's modulus and the systematic design of the circuit layout to distribute the stress concentration would intrinsically eliminate such interconnection problems and allow the complete assembly of devices for highly stretchable integrated chips.

## Methods

### Encapsulated thin metal electrode fabrication

The fabrication process begins with the generation of a sacrificial GeO_*x*_ (thickness=300 nm) layer and a protective SiO_2_ (thickness=100 nm) layer on a Si wafer chip using RF sputter (Ar/O_2_, 10/5 s.c.c.m., 60 W, 2.5 mtorr, Korea Vacuum Tech., Ltd) and plasma-enhanced chemical vapour deposition (Ar/N_2_O/SiH_4_, 100/30/15 s.c.c.m., 100 W, 2 torr, SNTEK), respectively. Coating a PI precursor (polyamic acid, Sigma-Aldrich) on the SiO_2_/GeO_*x*_/Si substrate at 3,000 r.p.m. followed by annealing at 250 °C for 2 h creates a PI substrate (thickness=2 μm). Cr/Au layers (thickness=5/70 nm) were deposited in sequence on the PI substrate using a d.c. sputter (Ar, 15 s.c.c.m., 290 V, 5 mtorr). Photolithography (Mask aligner: CA-6M, SHINU MST, illumination: 8.5 mW cm^−2^, photoresist (PR): GXR-601, 4,000 r.p.m., exposure time: 7 s) and wet etching (CR-7 OMG for Cr, TFA TRANSENE for Au) were used to form the patterned metal electrode. Coating PI precursor on the substrate followed by annealing at 250 °C for 2 h encapsulates the electrode to form a neutral mechanical plane.

### Cilia-assisted transfer printing process

To generate the cilia structure, the main substrate was protected by the deposition of a Cr film (thickness=50 nm) followed by a conventional patterning process (photolithography and wet etching). RIE (O_2_ flow rate of 20 s.c.c.m., 150 W, 50 mtorr, 7 min) on the unprotected PI region and removing the residual Cr mask by wet etching generated the desired thickness (1.8 μm) of the cilia zone. A second full etching process using a new Cr mask for the cilia completed the formation of the cilia structures. Coating the substrate with PMMA (MicroChem, 950K, A11) created a layer to provide mechanical support to the ultrathin layer during the transfer printing process. After etching the GeO_*x*_ sacrificial layer completely at 70 °C in water, the floated device was transferred onto a textile substrate, and the supportive PMMA layer was dissolved by PDMS solution (10:1 mixture of PDMS precursor and curing agent in toluene, Sylgard 184, Dow Corning). Finally, the devices on textile were dried by annealing at 120 °C for 2 h.

### Fabrication of IGZO-based 7-stage RO

After the generation of PI/SiO_2_/GeO_*x*_/Si substrate in the process for encapsulated thin metal electrode fabrication, an additional SiO_2_ buffer layer (thickness=100 nm) was deposited on the PI substrate to avoid significant thermal expansion of the plastic substrate and thereby allow a broad temperature range in fabrication. Next, Mo (thickness=70 nm) was deposited using a DC sputter (Ar, 15 s.c.c.m., 240 V, 5 mtorr) and patterned by a conventional photolithography and RIE process (SF_6_/O_2_ flow rate of 10/40 s.c.c.m., 100 W, 100 mtorr, 1 min) to form a gate electrode layer. SiO_2_ (thickness=150 nm) was deposited by plasma-enhanced chemical vapour deposition (Ar/N_2_O/SiH_4_, 90/100/10 s.c.c.m., 100 W, 2 torr) and patterned by photolithography and RIE (CF_4_/O_2_ flow rate of 50/2.5 s.c.c.m., 100 W, 100 mtorr, 3 min) to form a gate dielectric layer. After the deposition of the IGZO channel layer (thickness=15 nm) on the gate dielectric layer by RF sputtering (Ar/O_2_, 15/ 0.15 s.c.c.m., 50 W, 2.5 mtorr), Au/Cr layers (thickness=150/5 nm) were deposited and patterned by photolithography and a lift-off process for the source/drain electrodes and metal lines of the 7-stage ring oscillator. Coating and patterning of the diluted SU-8 solution (8 wt% SU-8 2010 precursor solution in SU-8 thinner, MicroChem) by conventional photolithography formed a passivation layer (thickness=100 nm) on the channel layer for electronic performance in a stable manner^44^. After encapsulation of the device by PI film, and RIE processing and applying silver paste for the external contact region, cilia-assisted transfer printing was conducted. For the durability test in wet conditions for laundry, the device was dipped in detergent solution (LG Household & Health Care Ltd., TECH, 0.06% by weight) for 20 min and rinsed in tap water for 30 min. After cleaning, the RO was dried at room temperature, followed by annealing at 120 °C on a hotplate.

### Characterization method

The measurement of electrical performance of the fabricated devices was conducted using an Agilent B1500A semiconductor parameter analyser (Agilent Technologies), a Keithley 2400/4200 (Keithley Instruments, Inc.) and a digital oscilloscope DSO7104 (Agilent Technologies). The adhesion and stretching test was performed using a digital force gauge (DS2-5N, IMADA Co, Ltd.) and a motorized linear actuator force gauge (LTS-HS, Newport). SEM, AFM, and optical microscope images were obtained using a Hitachi S-4700 microscope (Hitachi, Ltd.), Park Systems XE-100 (Park Systems Corp.) and BX51 system microscope (Olympus), respectively. Thermal imaging was performed by IR camera (FLIR A655sc, FLIR systems, Inc.). The surface of the PI film was analysed using XPS (AXIS-NOVA, Kratos. Inc.) and ATR-FTIR spectroscopy (VARIAN 660-IR, Varian. Inc.).

## Additional information

**How to cite this article:** Yoon, J. *et al*. Robust and stretchable indium gallium zinc oxide-based electronic textiles formed by cilia-assisted transfer printing. *Nat. Commun.* 7:11477 doi: 10.1038/ncomms11477 (2016).

## Supplementary Material

Supplementary Information Supplementary Figures 1-31, Supplementary Tables 1-5, Supplementary Notes 1-10 and Supplementary References

Supplementary Movie 1 To determine the contribution of the cilia to the interfacial adhesion between a transferred substrate and a target surface, a detachment test was conducted by blowing air on the 2 × 2 PI open square pattern arrays. No glue was used in the detachment test shown in this video. The substrates with cilia still maintain their positions at comparably higher air pressure compared to those with no cilia.
