# Supplementary material for: Robust and stretchable indium gallium zinc oxide-based electronic textiles formed by cilia-assisted transfer printing
Source: Nat Commun. 2016 Jun 1;7:11477. doi: 10.1038/ncomms11477 (PMC4895352; doi:10.1038/ncomms11477)
Supplement: Supplementary Information — Supplementary Figures 1-31, Supplementary Tables 1-5, Supplementary Notes 1-10 and Supplementary References [file ncomms11477-s1.pdf]

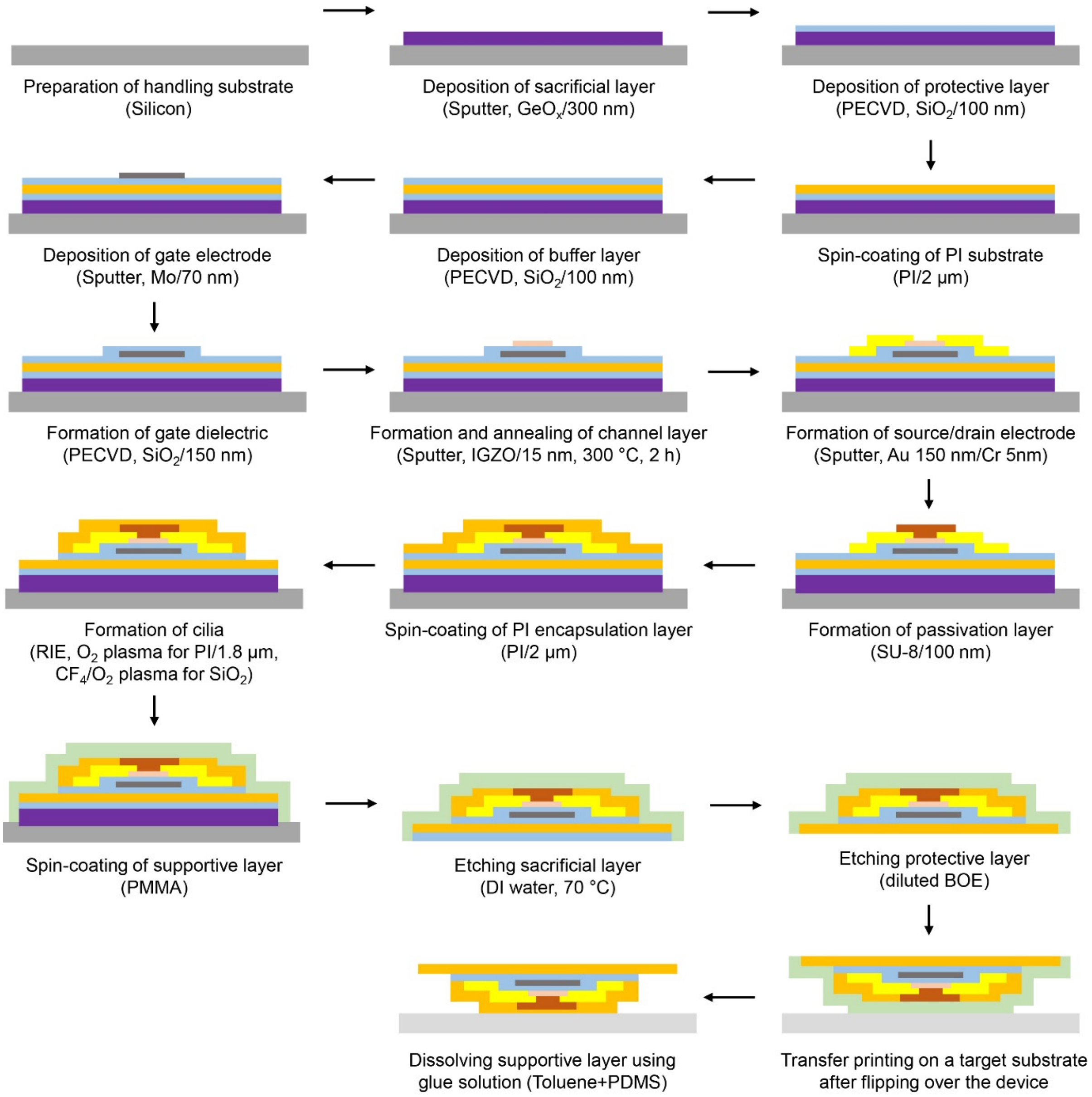

Supplementary Figure 1 | Fabrication flow of an IGZO-based transistor. See experimental section for detailed description.

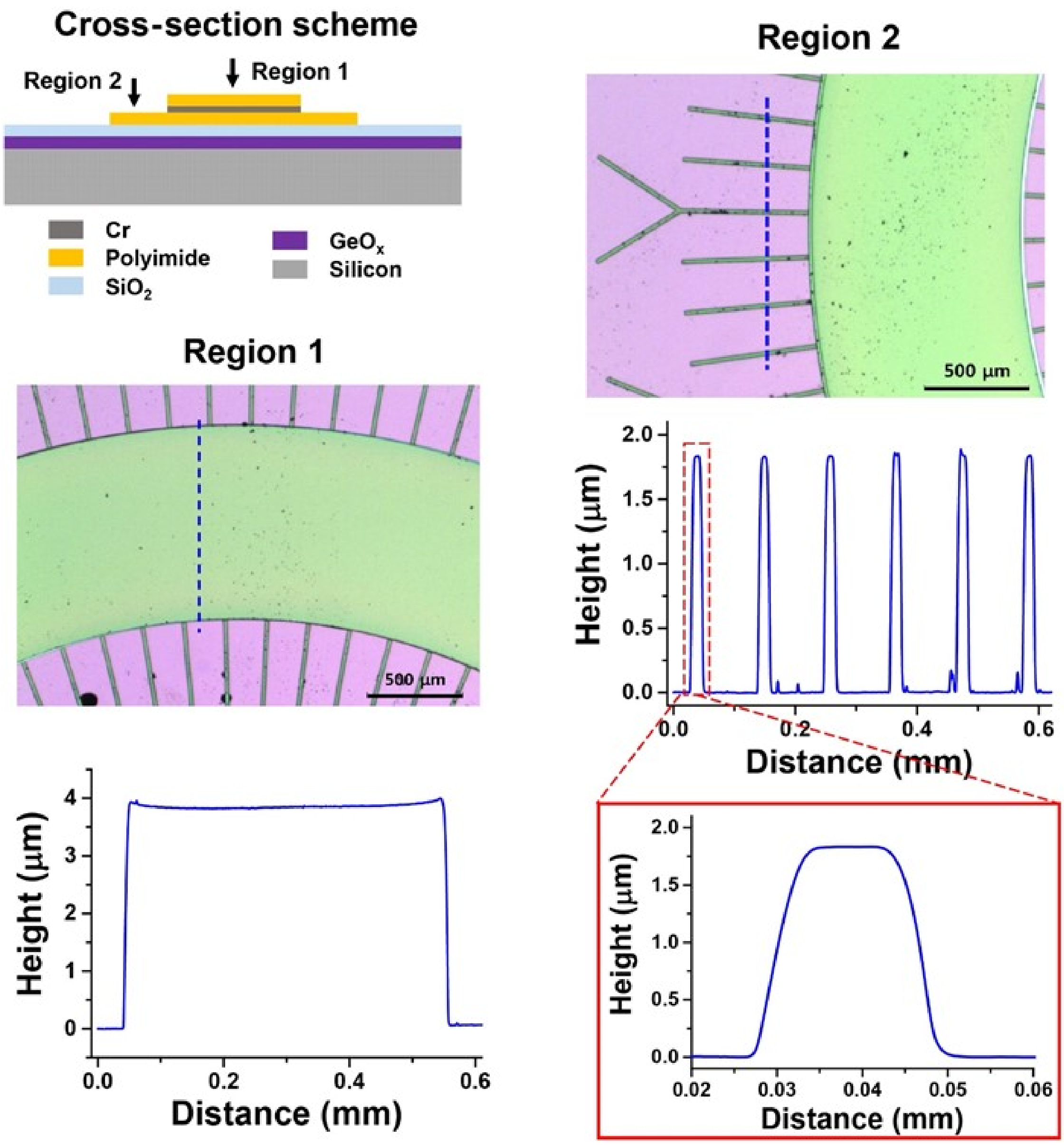

Supplementary Figure 2 | Schematic diagram and optical microscope images of main substrate [PI (2 μm)/Cr (70 nm)/PI (2 μm)] and cilia [PI (1.8 μm)]. Their thickness was measured by a surface profiler.

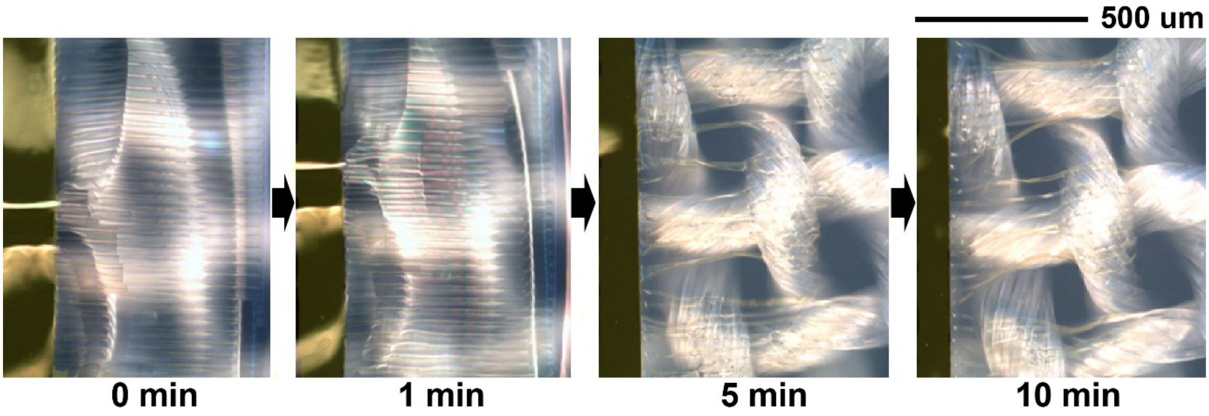

Supplementary Figure 3 | Wrapping process of cilia during dissolving process of PMMA supportive layer. Optical microscope images of the main substrate [PI (2 μm)/Cr (70 nm)/PI (2 μm)] and cilia observed during dissolving and drying process for 10 min. We used toluene to dissolve the supportive layer.

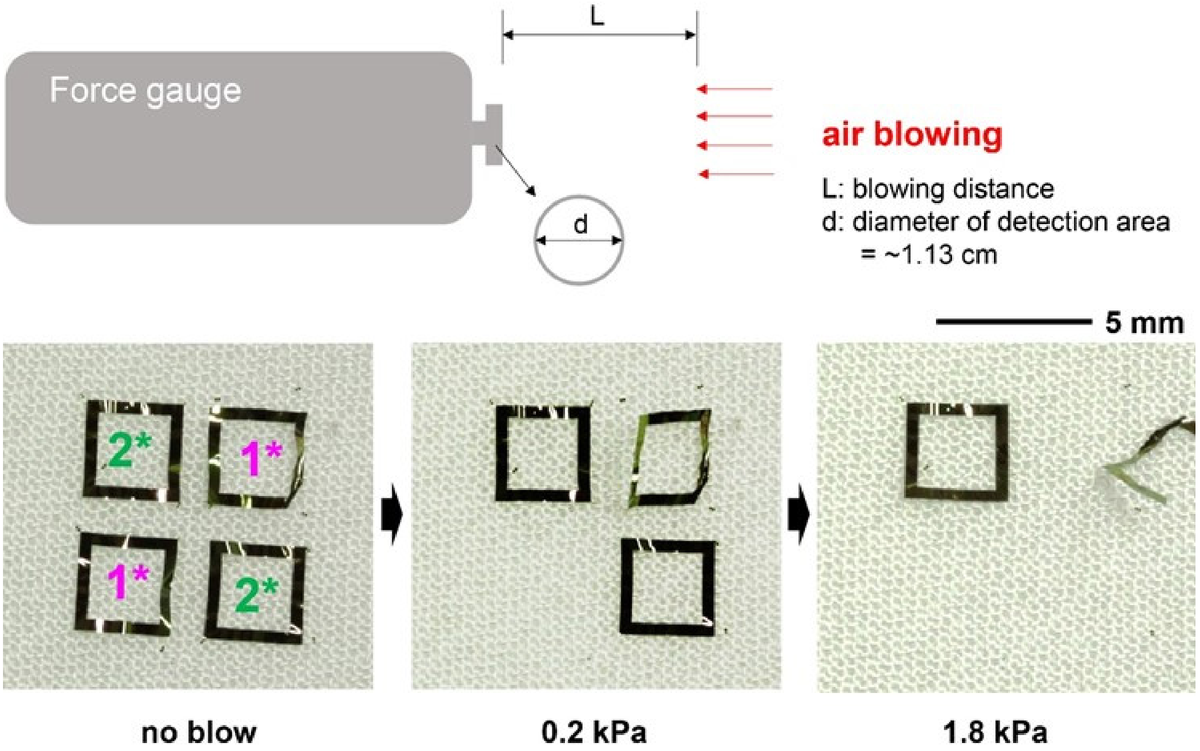

Supplementary Figure 4 | Detachment test of the main substrate [PI (2 μm)/Cr (70 nm)/PI (2 μm)] with (noted by 2*) and without cilia (noted by 1*). Set-up scheme for detachment test (top) and photographs of the samples after applying different blowing pressures.

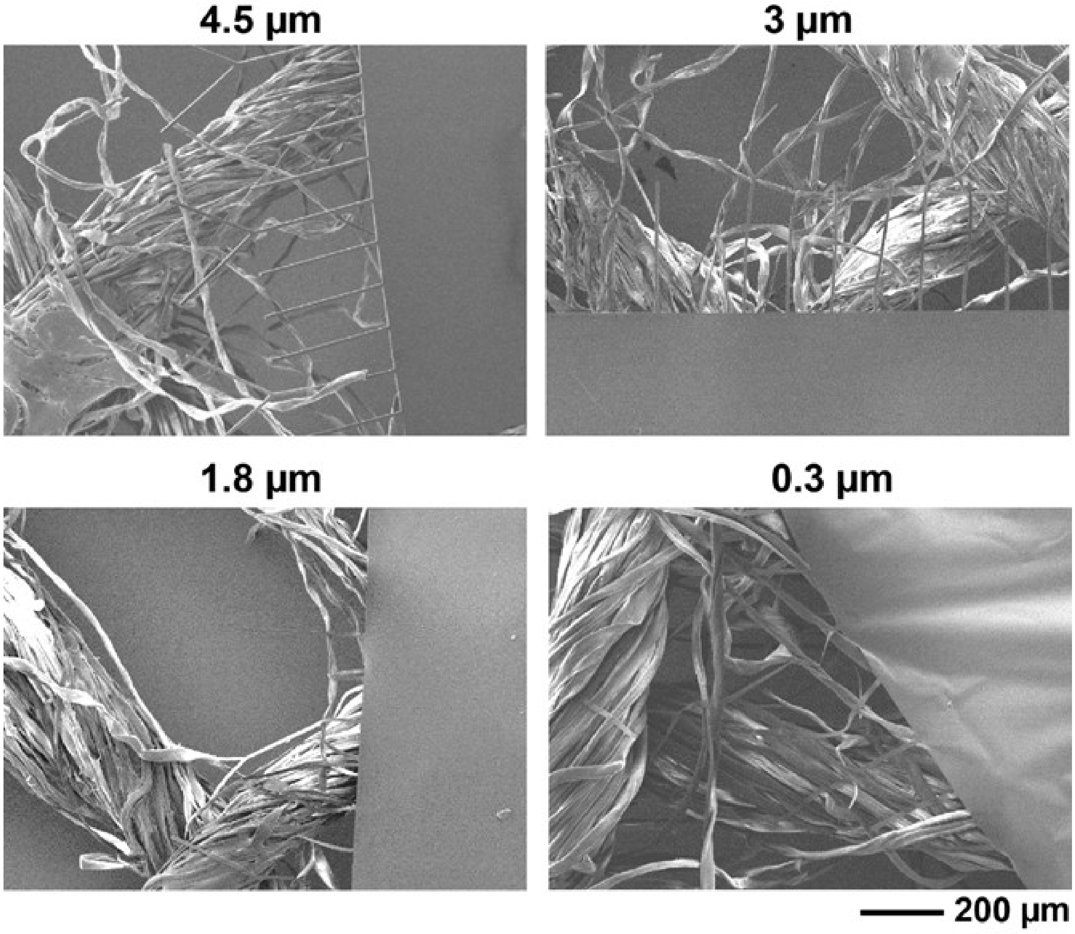

Supplementary Figure 5 l Stiffness and conformal wrapping of cilia with different thicknesses on threads. SEM images of the patterned PI films with different thicknesses (Thickness = 0.3, 1.8, 3 and 4.5 μm, respectively).

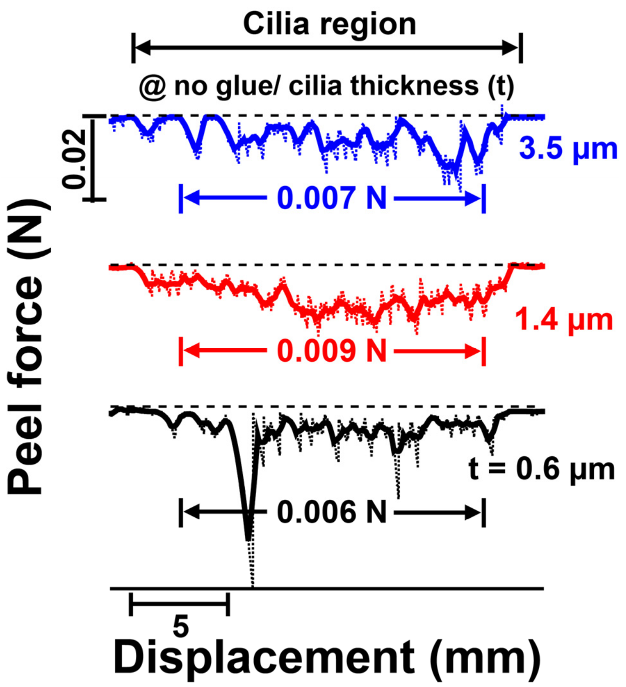

Supplementary Figure 6 | Peel test for the substrates using cilia with thicknesses of 3.5, 1.4 and 0.6 μm.

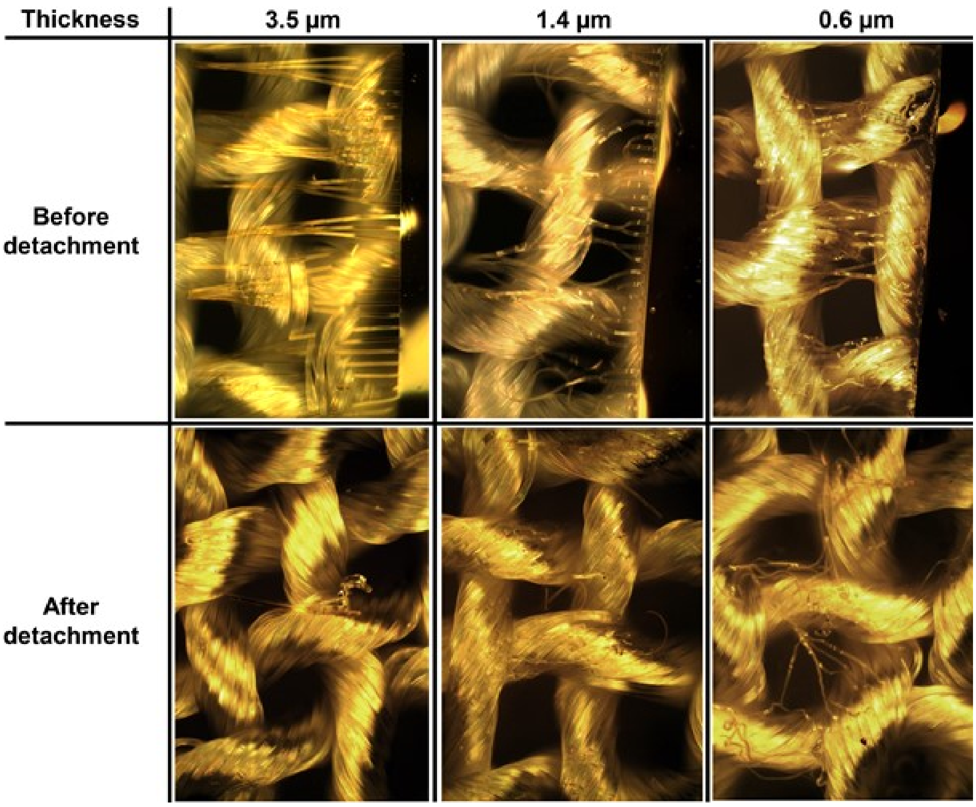

Supplementary Figure 7 | Dark-field optical microscope image for the substrates with various cilia thicknesses of 3.5, 1.4 and 0.6 μm before and after peel test.

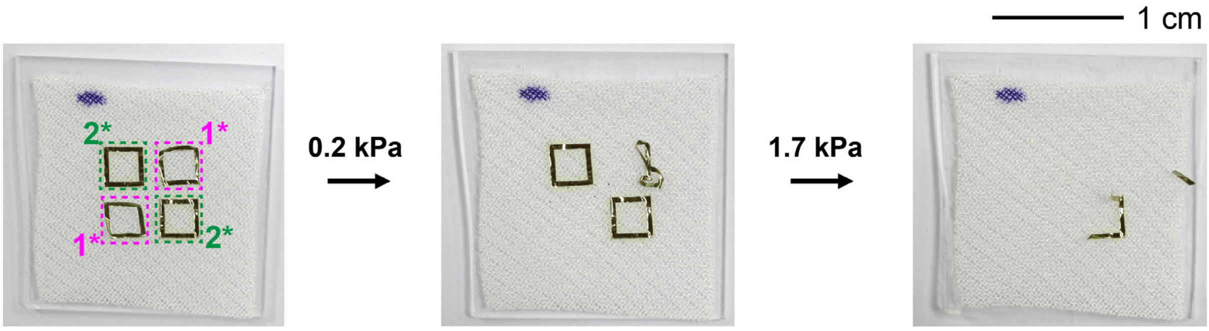

Supplementary Figure 8 | Detachment test for the main substrates flipped over in transfer printing process onto a textile. Photographs of the main substrates [PI (2 μm)/Cr (70 nm)/PI (2 μm)] with (noted by **2***) and without cilia (noted by **1***) on a textile substrate at different applied air pressures.

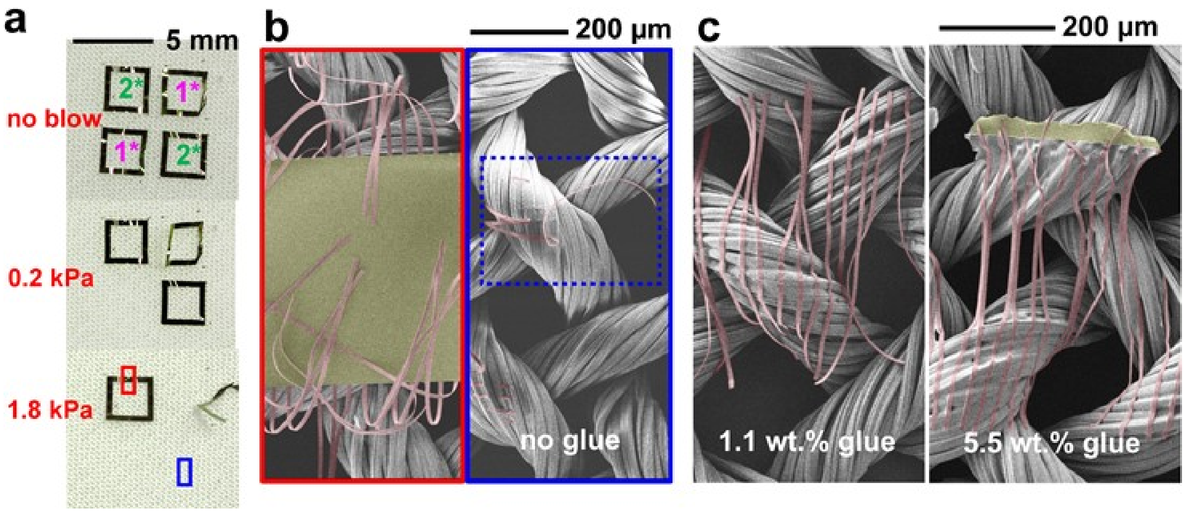

Supplementary Figure 9 | Photographs and SEM images after detachment test. **a**, Photographs of the main substrates [PI (2 μm)/Cr (70 nm)/PI (2 μm)] with (noted by **2***) and without cilia (noted by **1***) on a textile substrate at various applied air pressures. **b,c**, SEM images of the selected area in red and blue boxes after intensive air-blowing on the samples without glue (**b**) and with different concentrations of glue solution (**c**).

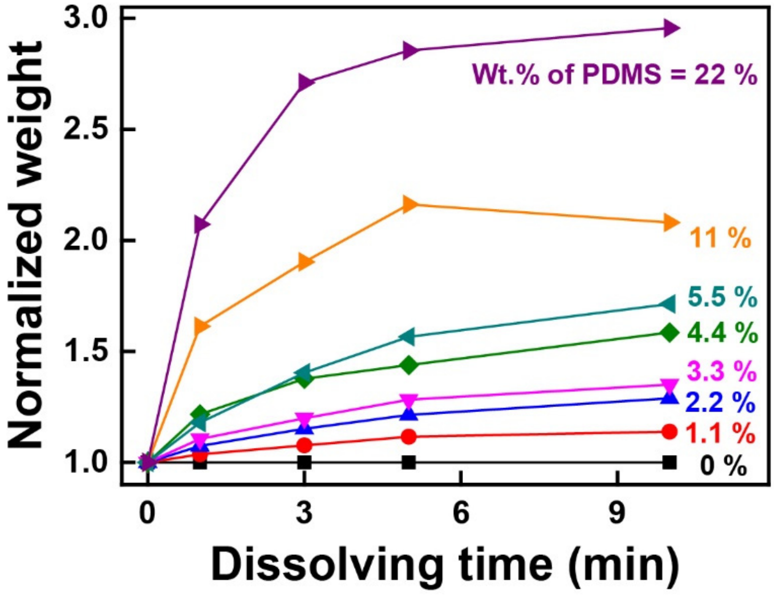

Supplementary Figure 10 | Normalized weight measured during dissolving process. The weight of the textile normalized to the main substrate [PI (2 μm)/Cr (70 nm)/PI (2 μm)] measured in the transfer printing process.

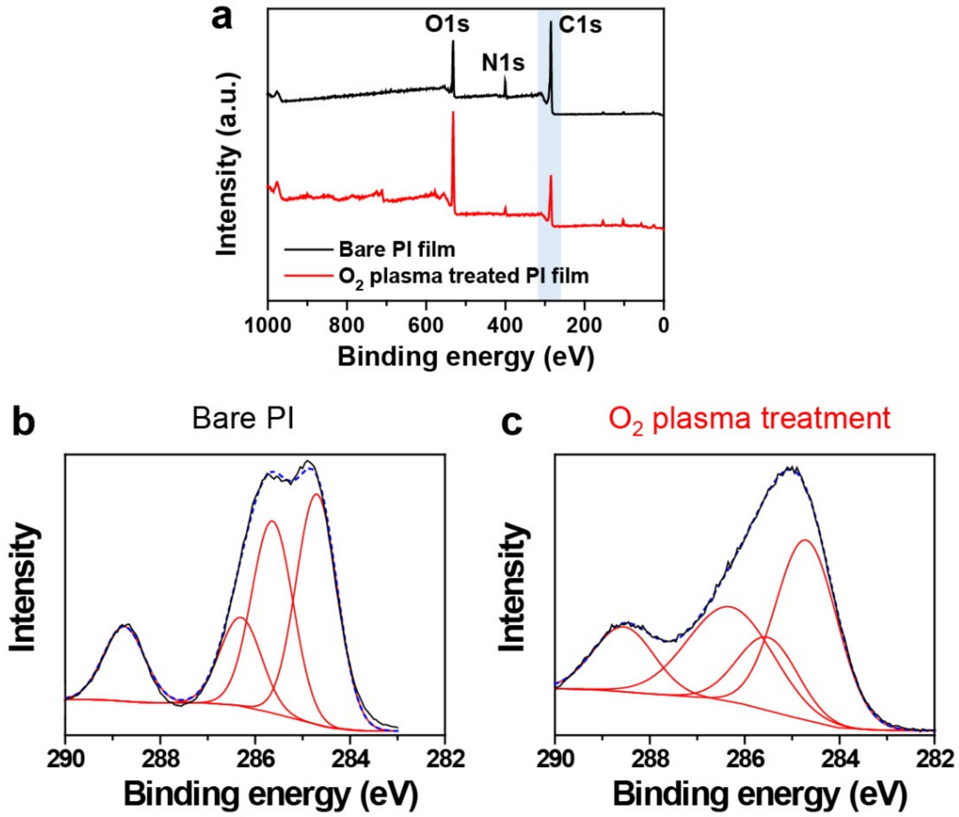

Supplementary Figure 11 | XPS analysis of the surface of PI film with and without O_2 plasma treatment for 7 min. Survey scan spectra **(a)** and C1s spectra **(b,c)** for the surface of the PI film with and without O_2 plasma treatment.

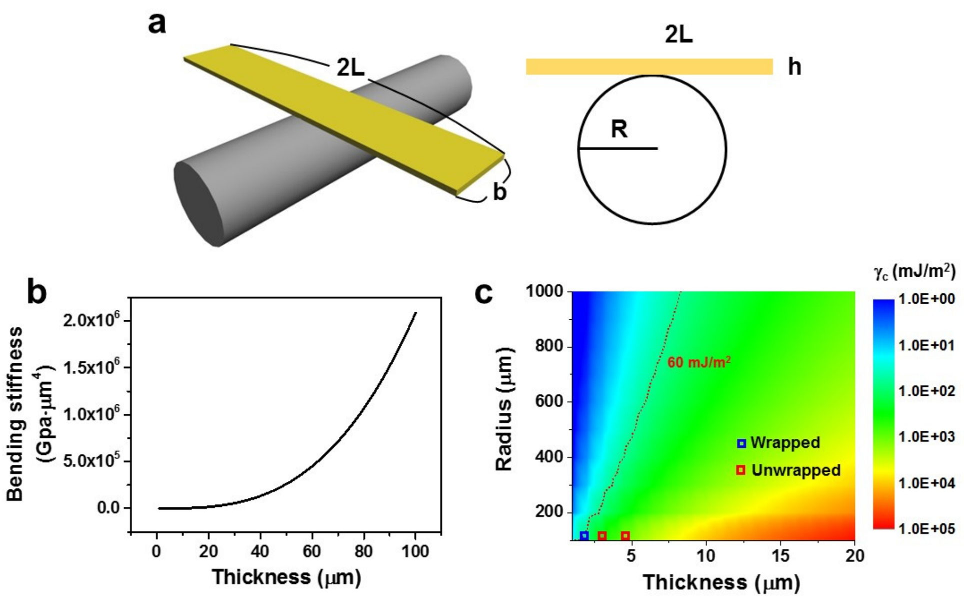

Supplementary Figure 12 | Calculated bending stiffness (EI) of a thin film with different thicknesses and the critical adhesion energy per unit area (γ_c) for conformal wrapping on a cylinder with radius of R. a, Schematic diagram of the thin film (2L: length, b: width, h: thickness) on a cylinder (R: radius). **b**, EI values as a function of the thickness of a thin film. **c**, Calculated γ_c values with different thicknesses of a thin film (h) and radii of a cylinder (R). The red dotted line represents the same γ_c values, and the blue and red open squares the show experimental data from Supplementary Figure 5.

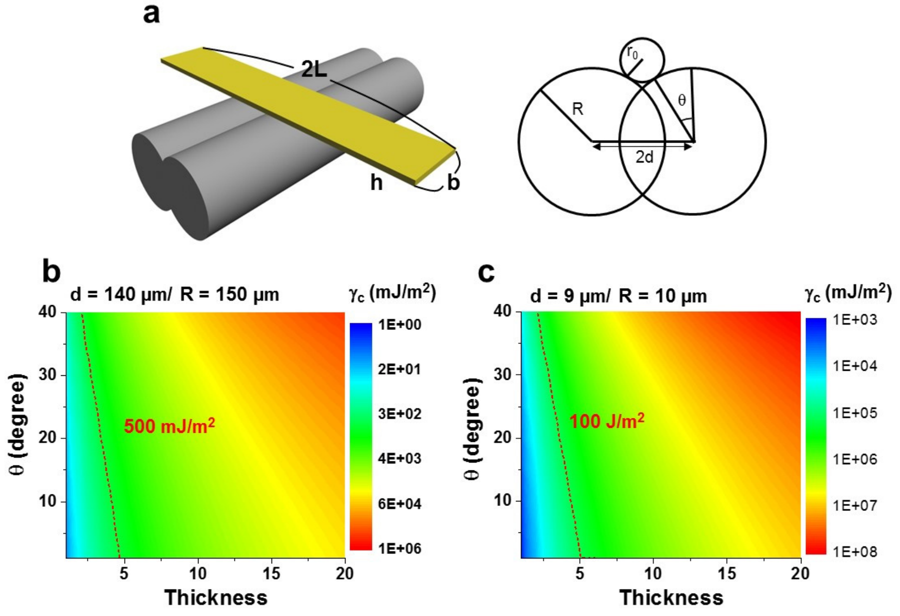

Supplementary Figure 13 | Critical adhesion energy per unit area (γ_c) for conformal wrapping of a thin film on two overlapped cylinders. a, Schematic diagram of the thin film on cylinders. **b and c**, Critical adhesion energy per unit area in two different cases.

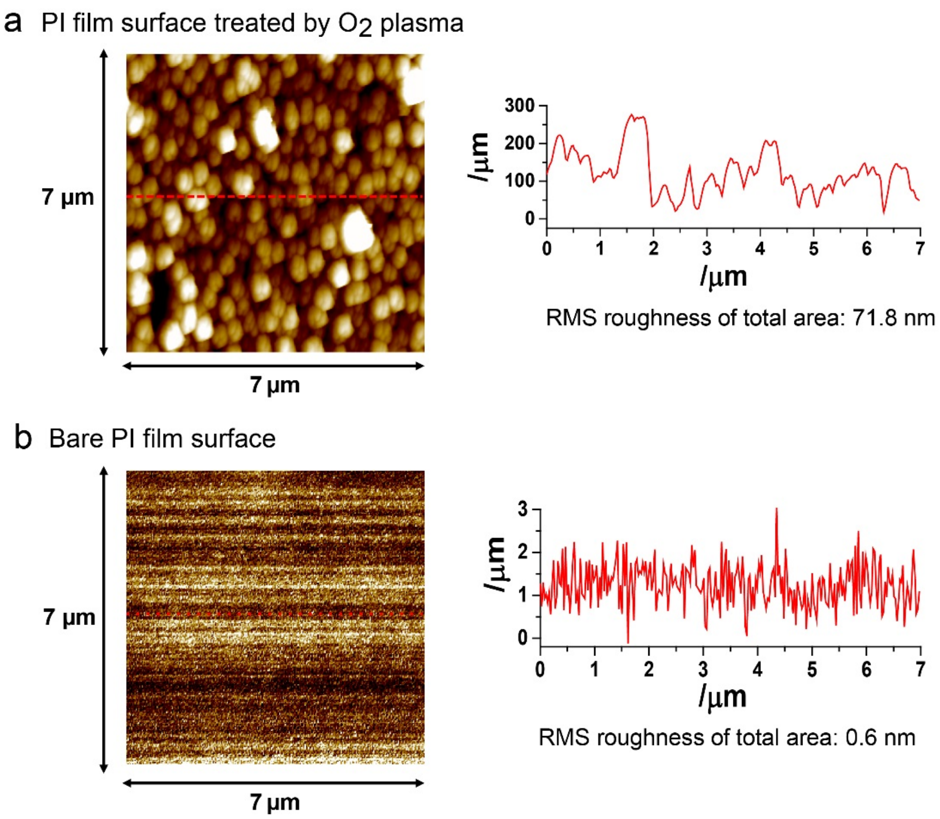

Supplementary Figure 14 | Surface morphology of PI films with and without O_2 plasma treatment for 7 min. AFM images and surface profiles of the PI films **(a)** with and **(b)** without O_2 plasma treatment.

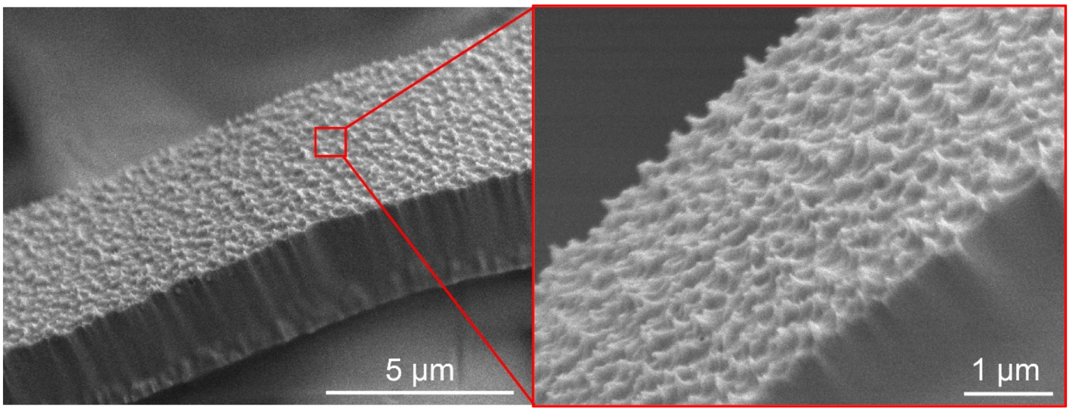

Supplementary Figure 15 | Thinned cilia with roughened surface induced by O_2 plasma. SEM image of cilia thinned by O_2 plasma treatment for 7 min (left) and magnified SEM image (right).

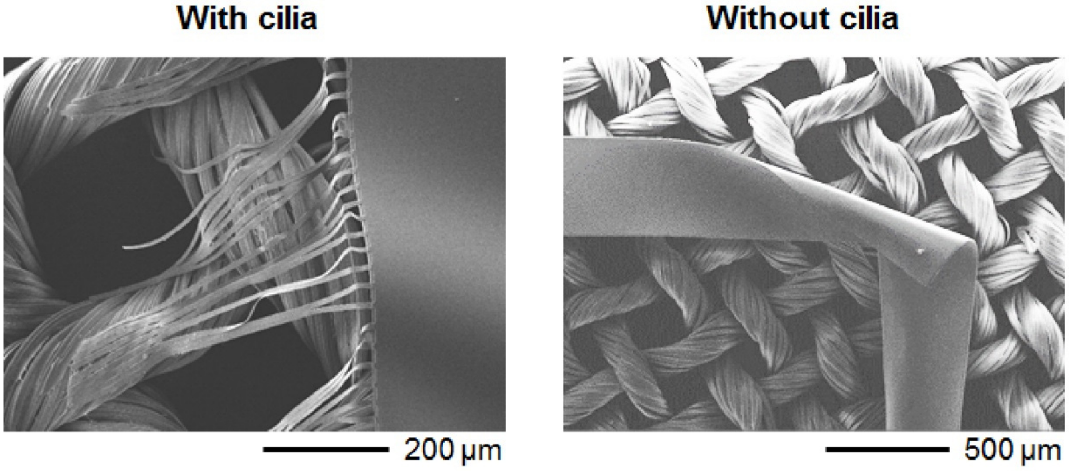

Supplementary Figure 16 l Undesirable deformation of the main substrate [PI (2 μm)/Cr (70 nm)/PI (2 μm)] and cilia when transferred onto a textile right side up. The SEM images of cilia (left) and the patterned substrate (right) transferred onto a textile substrate right side up.

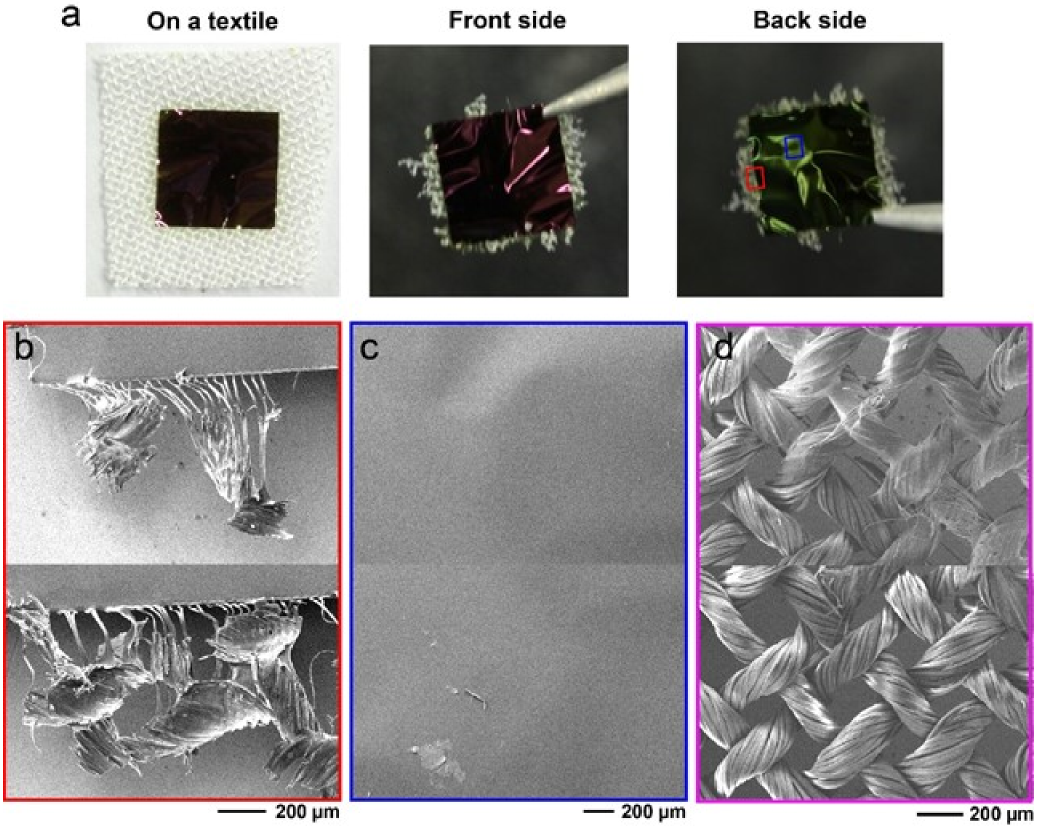

Supplementary Figure 17 | Distribution of residual glue after transfer printing. **a,** Photographs of the main substrate [PI (2 μm)/Cr (70 nm)/PI (2 μm)] on a textile (left) and front (centre)/back (right) side of the substrate detached by tweezers. **b,** SEM images for the edge of the detached substrate. The residual glue was concentrated near the cilia. **c,** SEM images for the back surfaces of the detached substrate. The residual glue was limited. **d,** SEM images of textile surface after detachment of the encapsulated electrode.

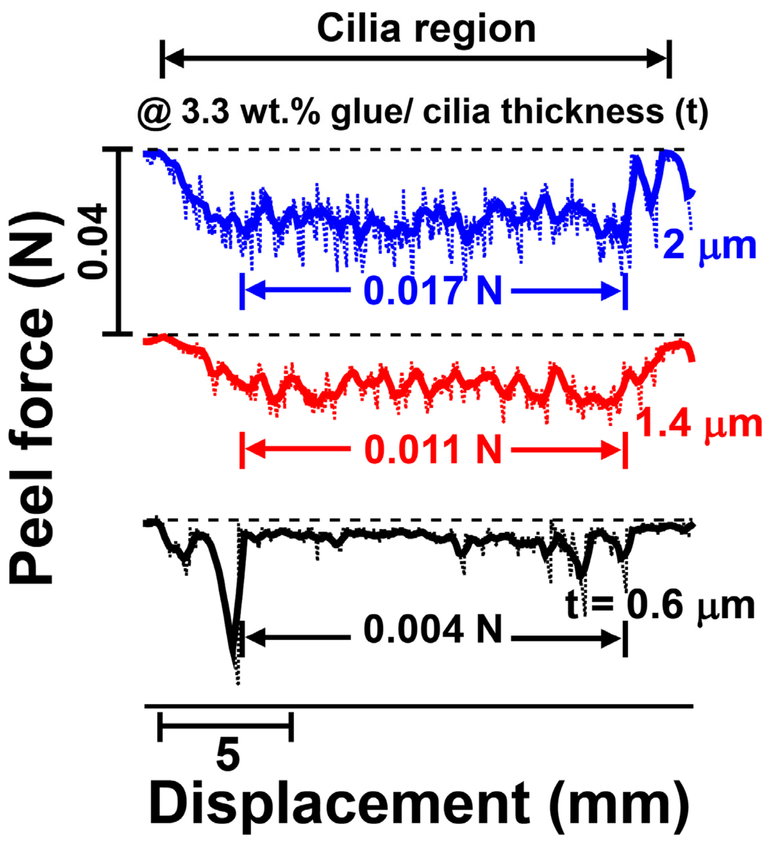

Supplementary Figure 18 | Peel test for the samples with cilia thicknesses of 0.6, 1.4 and 1.8 μm using 3.3 wt.% glue.

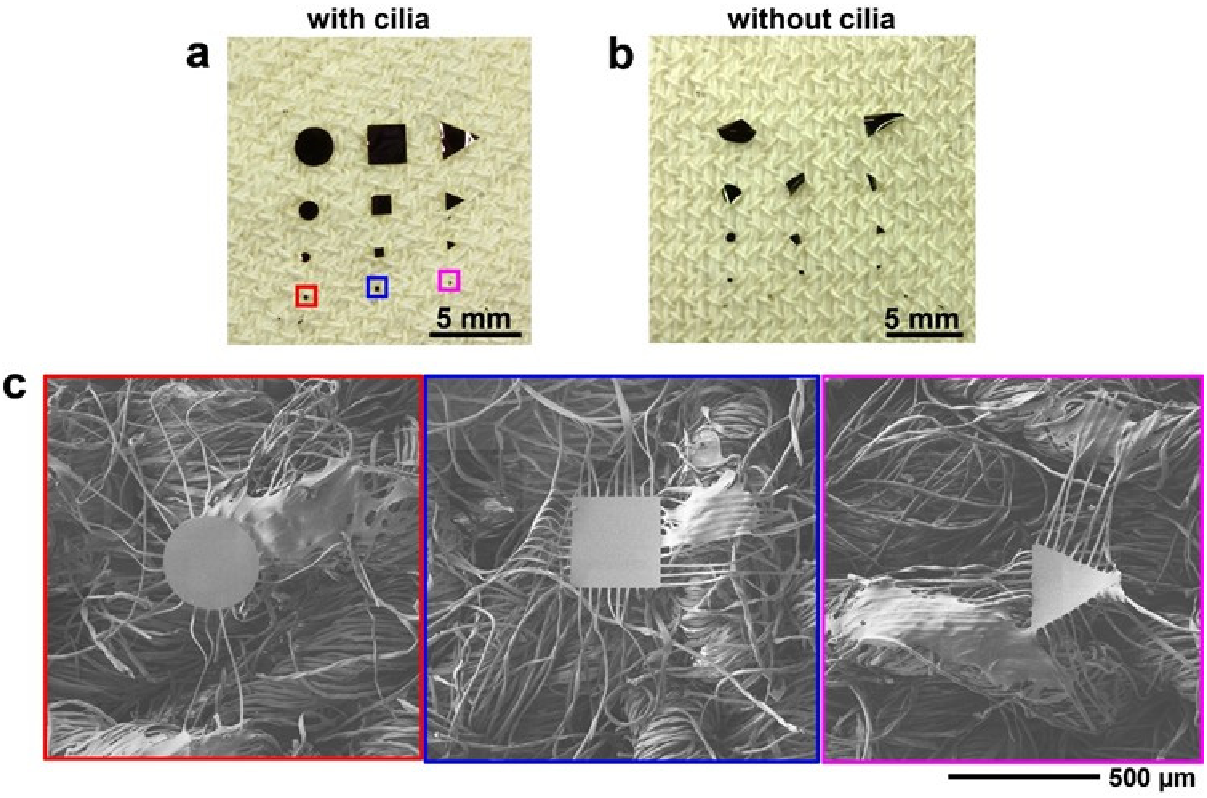

Supplementary Figure 19 | Transfer printing of the patterned substrates [PI (2 µm)/Cr (70 nm)/PI (2 µm)] with different sizes and shapes onto a bandage. Photographs of the transferred substrates **(a)** with and **(b)** without cilia. **c,** SEM images for the smallest patterns marked in **(a)**.

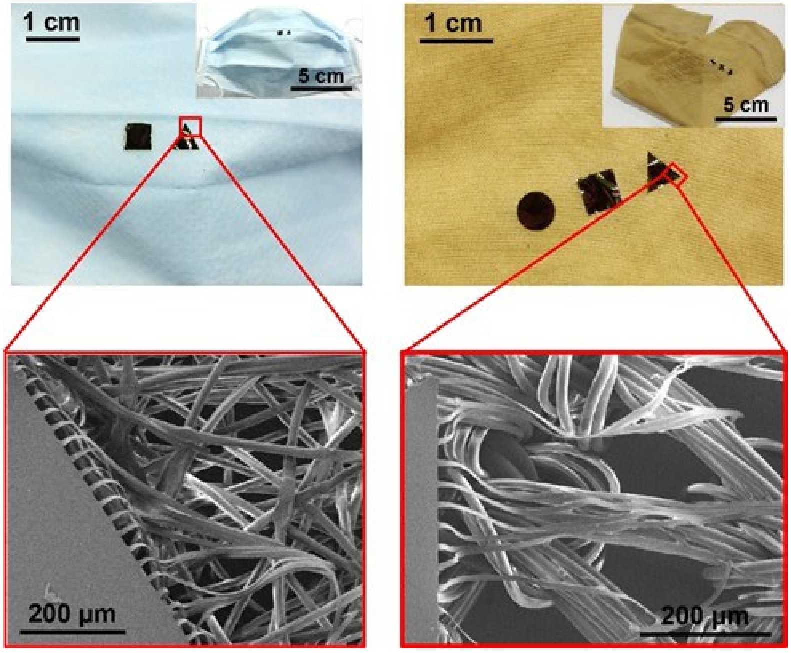

Supplementary Figure 20 | Examples of cilia-assisted transfer printing. Photographs and SEM images of the patterned substrates [PI (2 μm)/Cr (70 nm)/PI (2 μm)] with cilia transferred onto a mask (left) and a stocking (right).

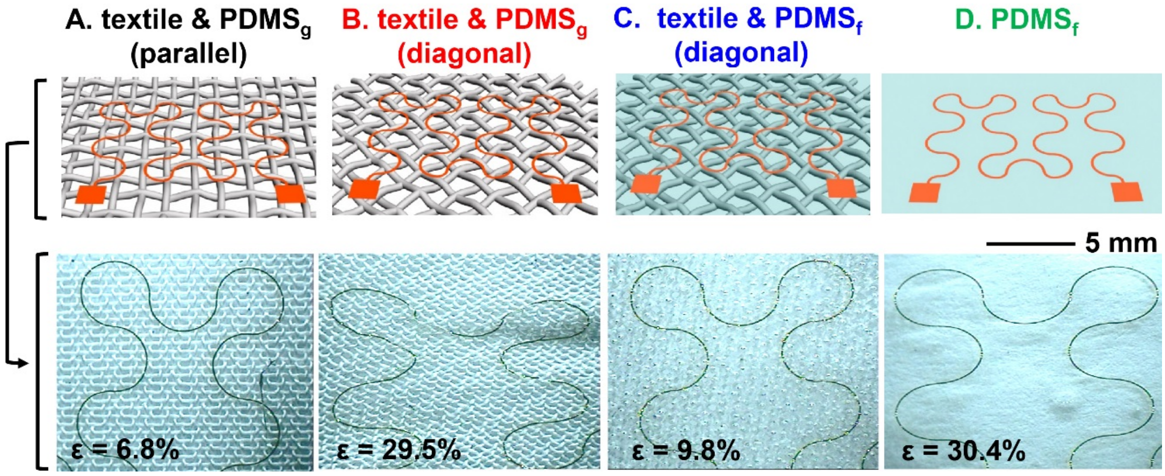

Supplementary Figure 21 | Encapsulated electrodes [PI (2 μm)/Au (70 nm)/Cr (5 nm)/PI (2 μm)] for stretching test in Figure 5. Schematic diagrams and optical microscope images of the encapsulated electrodes on a textile in parallel (A) and diagonal (B) directions and the encapsulated electrode on a textile embedded using PMDS film (C) and the electrode embedded using PDMS film only (D).

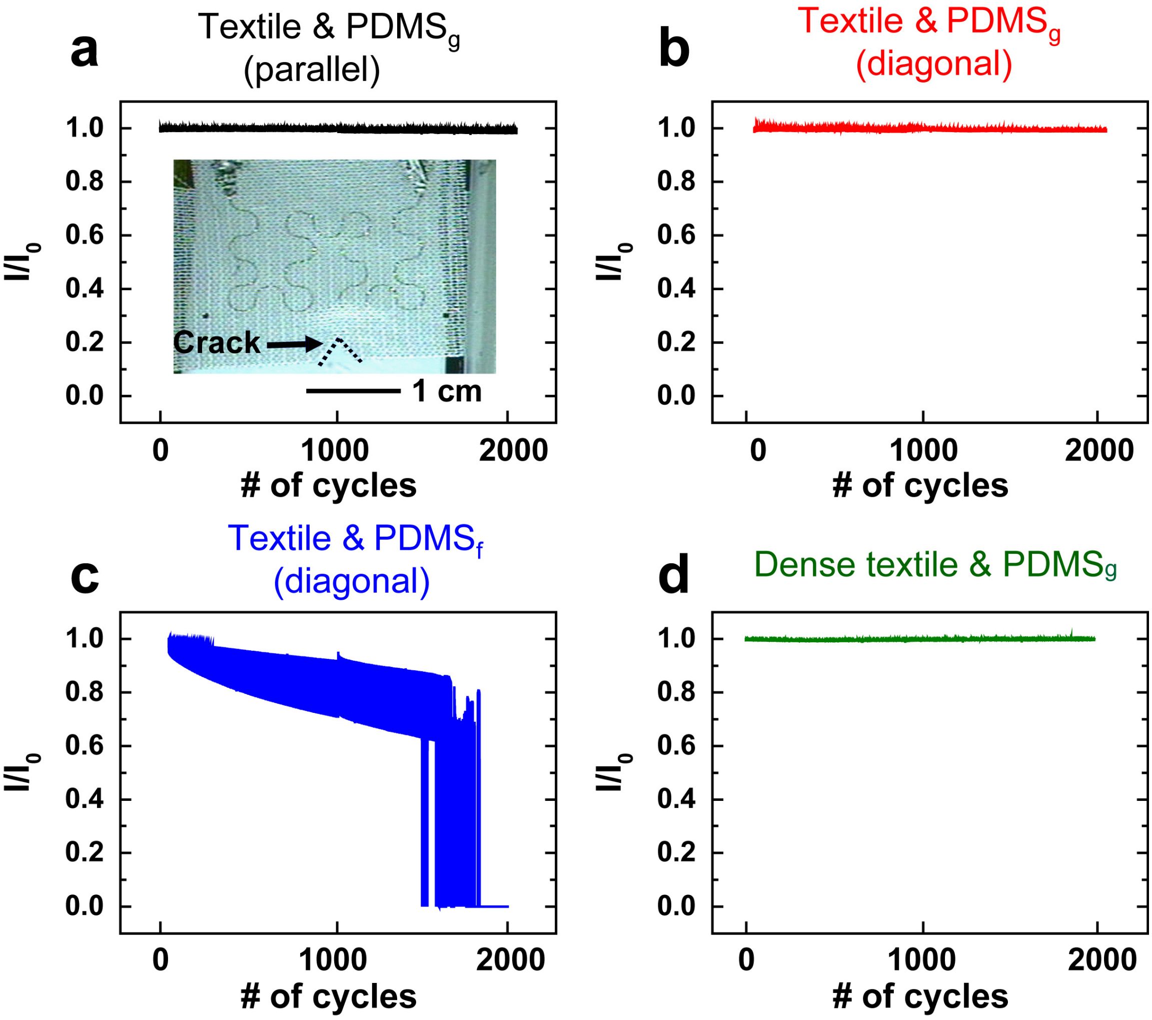

Supplementary Figure 22 | I/I_0 of the encapsulated electrodes [PI (2 μm)/Au (70 nm)/Cr (5 nm)/PI (2 μm)] upon repeated stretching and releasing corresponding to tensile forces of 0 and 3.5 ± 0.5 N for 2000 cycles. I/I_0 of the electrode transferred on a textile in parallel (a) and diagonal (b). The inset shows the representative optical microscope image of the sample with a cut by scissors at the edge of the textile generated after 1000 cycles; I/I_0 of the electrode on a textile embedded using PMDS film (c). **d,** I/I_0 of the electrode transferred onto a dense textile in the diagonal direction. Note that we generated a scratch in each film after 1000 cycles and continued additional measurements.

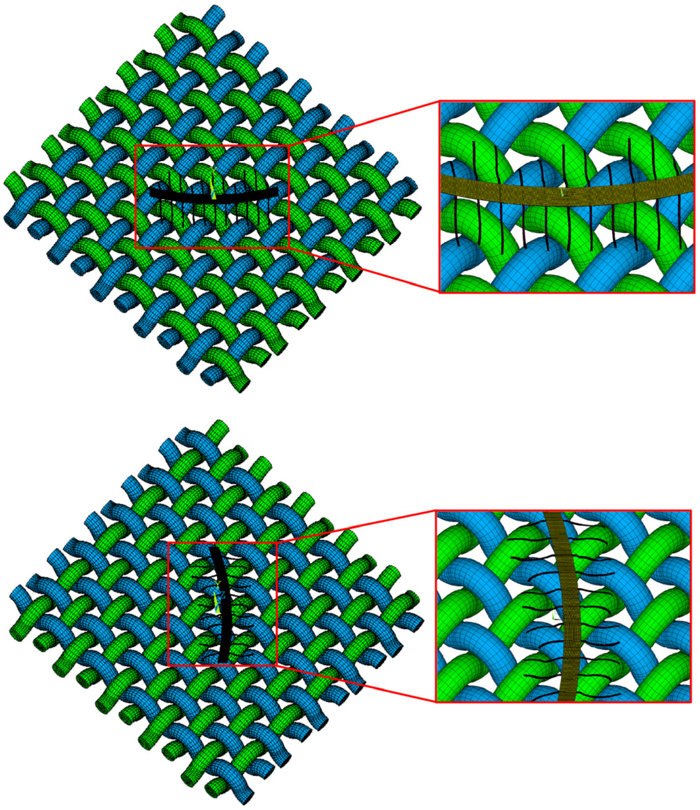

Supplementary Figure 23 | FEM Modeling

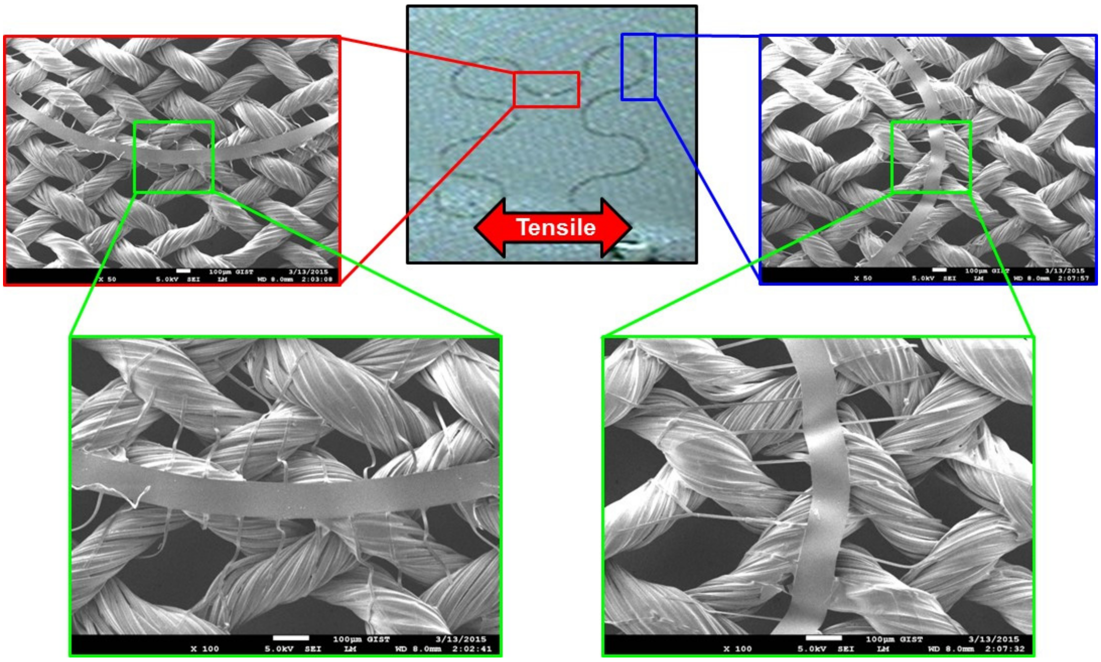

Supplementary Figure 24 | Optical microscope and SEM images used in FEM modelling.

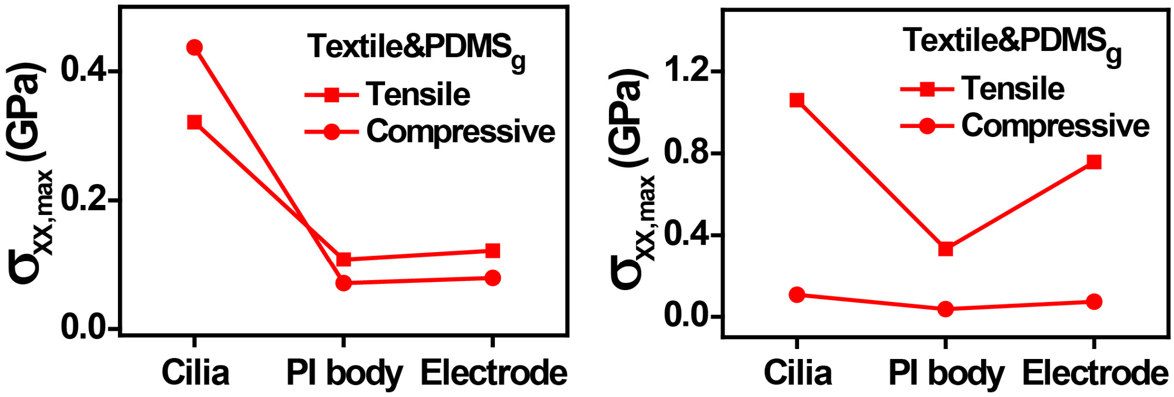

Supplementary Figure 25 | Maximum stress values for cilia, PI body, and electrode in encapsulated electrode [PI (2 μm)/Au (70 nm)/Cr (5 nm)/PI (2 μm)] at tensile strain = 9% for the electrode placed on the textile parallel (left) and perpendicular (right) to the stretching direction.

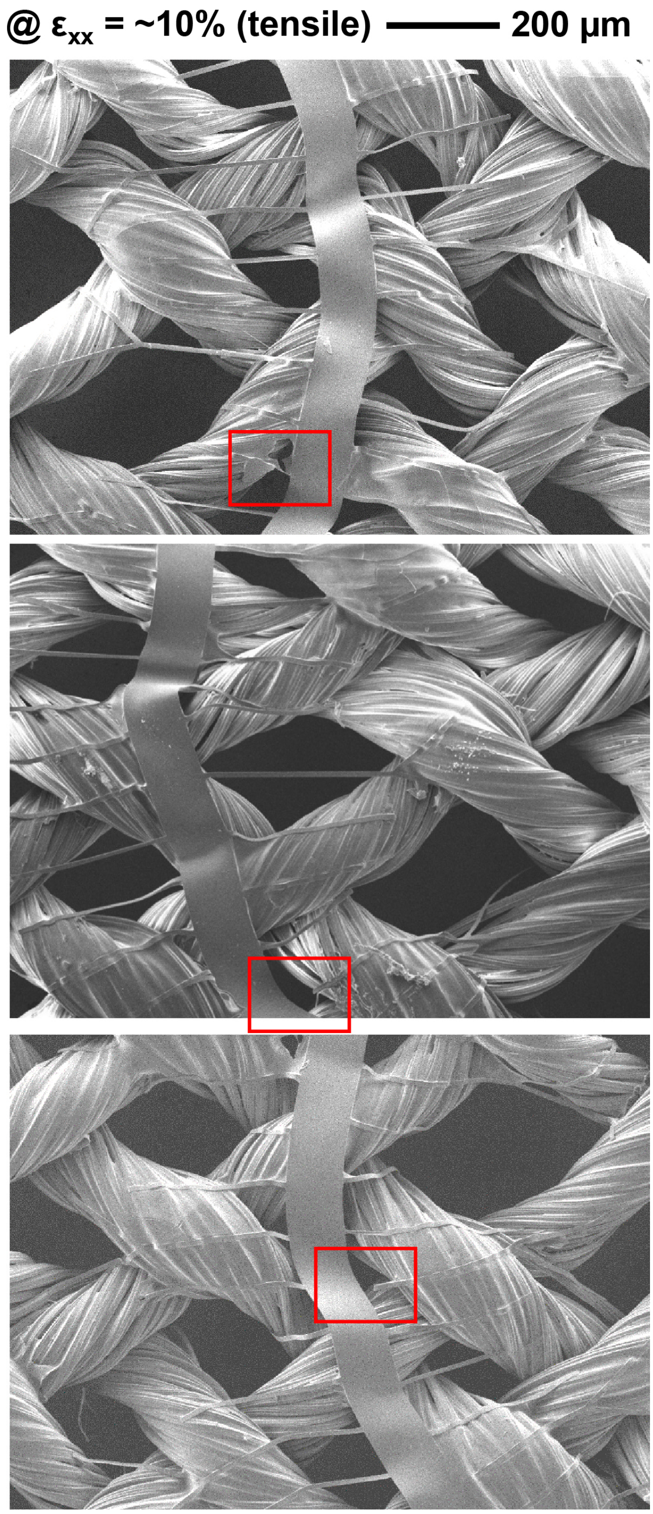

Supplementary Figure 26 | SEM images of cilia disconnected from the main PI body [PI (2 μm)/Au (70 nm)/Cr (5 nm)/PI (2 μm)] by excessive tensile stress (tensile strain = 10%).

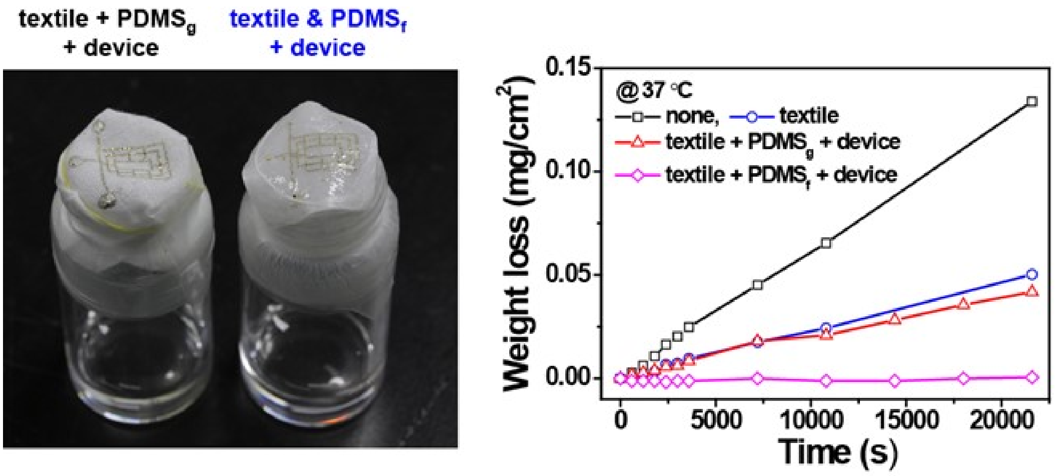

Supplementary Figure 27 | Water permeation test. a, Photograph of the prepared samples. **b,** Weight loss per unit area of water in vials without a cover (black line), and with only textile (blue line), textile/$PDMS_g$/device (red line) and textile/$PDMS_f$/device (magenta line) at 37 °C. In the open area of a vial, 20% of the textile was covered with the PI membrane.

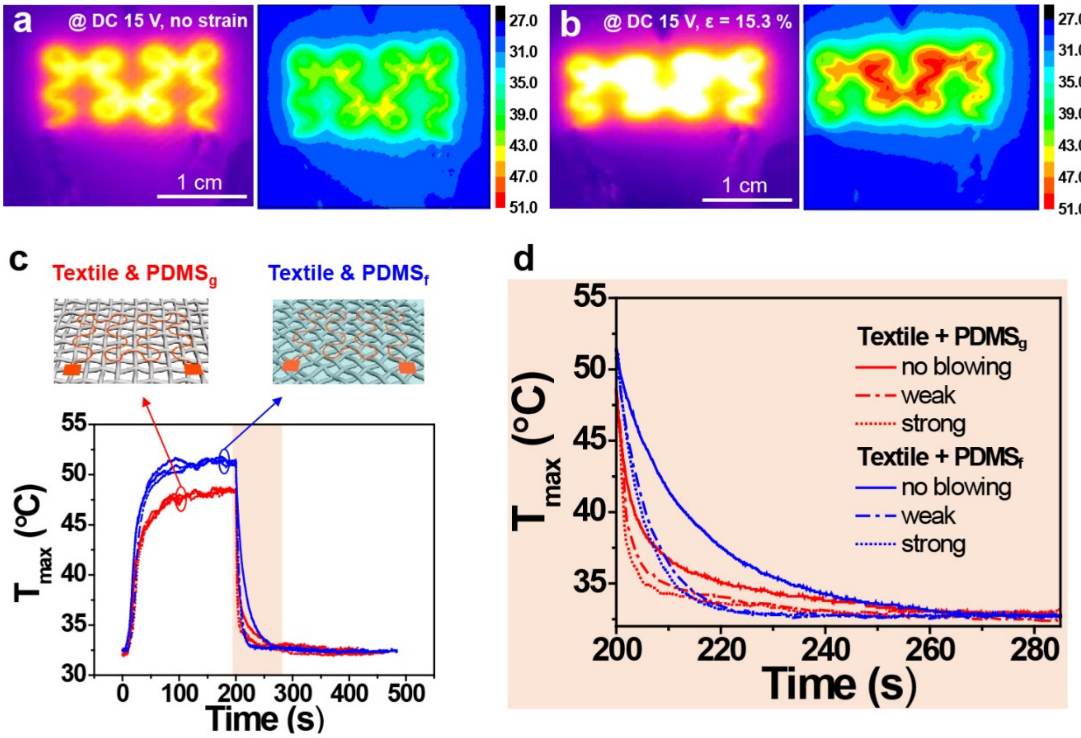

Supplementary Figure 28 | Heat dissipation characteristics of the encapsulated electrodes [PI (2 μm)/Au (70 nm)/Cr (5 nm)/PI (2 μm)] with textile & $PDMS_g$ and with textile & $PDMS_f$. Infrared camera images and temperature profiles for the electrode observed at DC 15 V with **(a)** no strain and **(b)** tensile strain ε = 15.3 %. **c,** Maximum temperature profile as a function of time for the electrode. We applied DC voltage from 0 to 15 V. **d,** Magnified region from (c) indicating the cooling of the electrode.

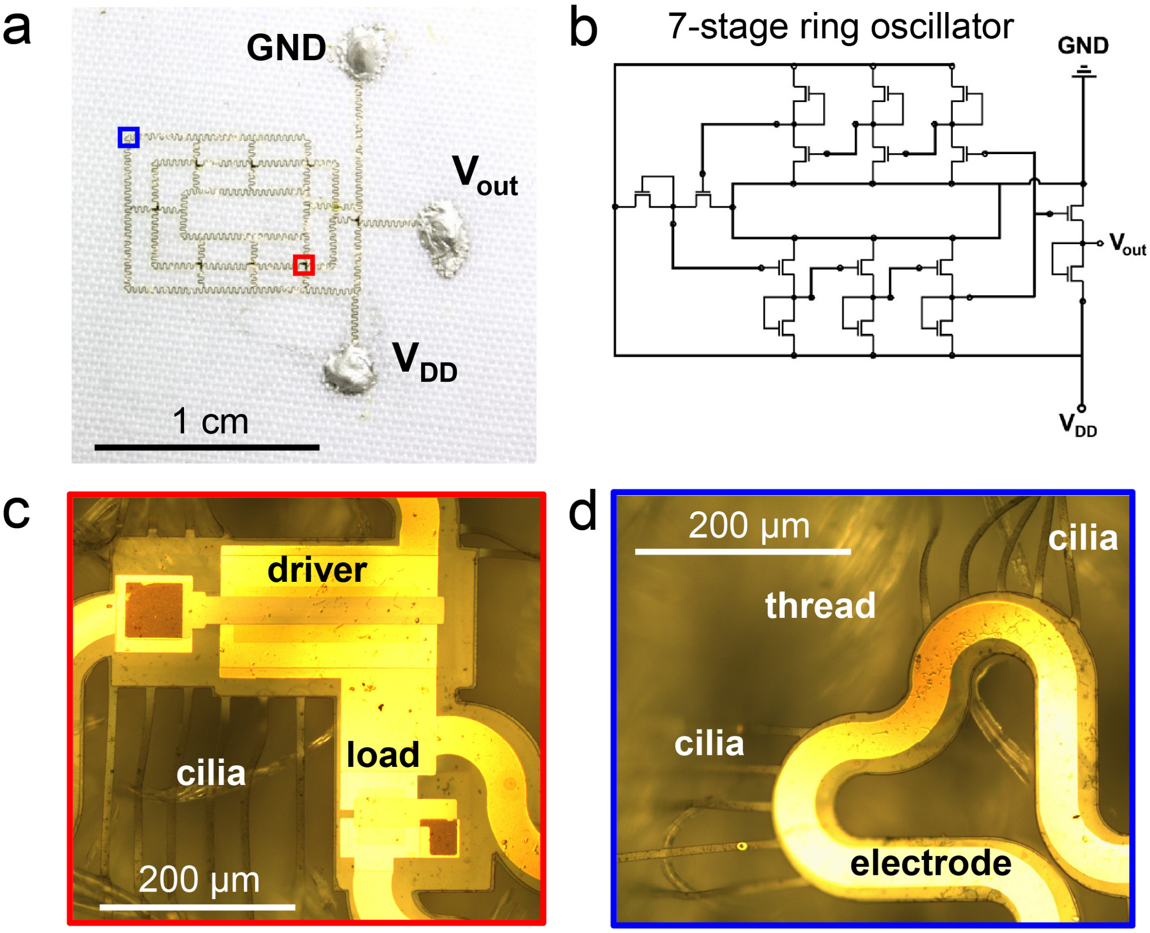

Supplementary Figure 29 | Photograph (a), equivalent circuit and optical microscope images (c and d) of the 7-stage ring oscillator.

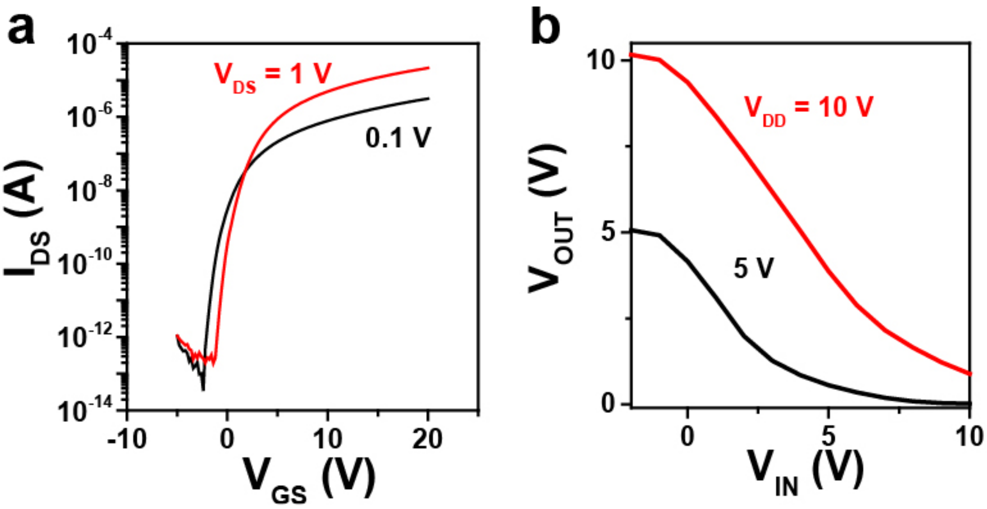

Supplementary Figure 30 l Basic electrical properties of the representative IGZO thin film transistor (TFT) and inverter. a, Transfer characteristics (I_{DS}-V_{GS}) of the IGZO TFT. **b,** Voltage transfer characteristics (V_{out}-V_{in}) of the inverter.

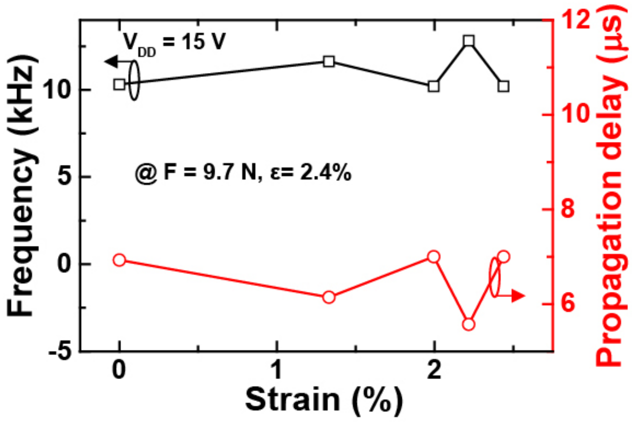

Supplementary Figure 31 l Stretching test for IGZO-based 7-stage ring oscillator transferred onto a textile in parallel direction. Frequency and propagation delay of the IGZO-based 7-stage ring oscillator measured with the application of tensile strain.

Supplementary Table 1 | Comparison of the critical pressures between the main substrates [PI (2 μm)/Cr (70 nm)/PI (2 μm)] transferred onto a textile right side up and upside down.

	Critical pressure (kPa)	
	Right side up	Upside down
With cilia	1.7	1.93
Without cilia	0.2	0.19

Supplementary Table 2 | Material properties used in this study.

Material	Young's modulus (GPa)	Shear modulus (GPa)	Poison's ratio (v)
Textile (rayon)	E_L : 18.3<br>E_T : 13.3	G_{TT} : 4.1<br>G_{TL} : 2.3	0.3
Polyimide	2.5	-	0.34
Au	75	-	0.34
Cr	275	-	0.28
PDMS	0.0018	-	0.48

E_L: Longitudinal modulus in the longitudinal direction

E_T: Transverse modulus in the transverse direction

G_{TL}: Longitudinal shear modulus in the longitudinal direction

G_{TT}: Transverse shear modulus in the transverse plane

Supplementary Table 3 | Slopes extracted from graphs in Supplementary Figure 19. The values show the permeability of each sample to water vapour.

	None	Textile	Textile + $PDMS_g$ + Device	Textile + $PDMS_f$ + Device
Slope ($\times 10^{-6}$) ($mg \cdot cm^{-2} \cdot s^{-1}$)	6.2	2.3	1.9	0.053

Supplementary Table 4 | Time constant values extracted from temperature decay curves in Supplementary Figure 28d.

Fan speed	Time constant, τ (s)/ thermal decay time, t (S)		
	Textile + $PDMS_f$	Textile + $PDMS_g$	
	τ_1 / t	τ_1 / t	τ_2
No	15.5 / 13.0	2.5 / 23.9	25.4
Weak	6.3 / 6.0	1.7 / 10.0	23.2
Strong	5.2 / 4.0	1.3 / 8.8	15.1

Supplementary Table 5 | Thermal conductivity and specific heat capacity of materials[1].

	Au	Cr	Polyimide	PDMS	Air	Cellulose (cotton)
Thermal conductivity ($W{\cdot}m^{-1}{\cdot}K^{-1}$)	315	69.1	0.12	0.15	0.024	0.23
Specific heat capacity ($J{\cdot}Kg^{-1}{\cdot}K^{-1}$)	129	461	1090	1460	1005	1300-1500

Supplementary Note1: Detachment test

We obtained the critical pressure using the measurement set-up shown in Supplementary Figure 4. We applied compressed air, which was calibrated using a force gauge, on prepared substrates and recorded the critical pressure when the substrates were detached from the textile (Supplementary Figure 4-bottom). The supplementary movie with a pinwheel, which is only for demonstration, intuitively shows that the substrates without cilia are only detached by blowing the compressed air.

Supplementary Note 2: Peel test for the substrates with different cilia thicknesses

We measured the peel forces for the substrates with various cilia thicknesses of 3.5, 1.4 and 0.6 μm. The average peel forces of the cilia regions were 0.007, 0.009, and 0.006 N, respectively (Supplementary Figure 6), with the peel force of the sample with 1.4 μm-thick cilia being the highest. We also examined the substrates before and after the peel test by optical microscope (Supplementary Figure 7) to determine the effect of the cilia thickness on the adhesion force. The relatively thin cilia (thickness = 0.6 or 1.4 μm) are flexible enough to allow conformal contact with the textile, whereas the thick cilia (thickness = 3.5 μm) are stiff without being bent before the peel test. On the other hand, the thin cilia (thickness = 0.6 μm) is torn more easily during the peel test than the thicker cilia (thickness = 1.4 or 3.5 μm). As a result of the mixed effect of the cilia thickness on the flexibility and mechanical strength, we believe that the optimal thickness for maximum overall adhesion is approximately 1.4 μm.

Supplementary Note 3: SEM images after detachment test

Supplementary Figure 9a shows photographs of the substrates after the detachment test using an air gun. The samples with cilia were detached at a higher air pressure. In the case of only cilia with no glue, most of the cilia were unwrapped from the threads upon intensive air-blowing over the critical pressure (Supplementary Figure 9b). On the other hand, when using both cilia and glue prepared with a PDMS precursor solutions of not less than 3.3 wt.%, the cilia were torn under intensive air-blowing (Supplementary Figure 9c). These results suggest that the realistic adhesion between the cilia and textile is much higher than the measured critical pressure when using glue.

Supplementary Note 4: The origins of adhesion between textile and cilia

The origin of the increase in P_c should be considered in terms of physical, chemical, geometrical and mechanical aspects. The first concerns the gravimetric loading effect of the ultrathin PI substrate. For all the specimens used in Fig. 2a and 2b, even level with the ground, the pressures required to eliminate the loading effect of the main substrate and cilia are only 0.41 Pa and 0.72 Pa, respectively, corresponding to 0.21% and 0.36% of P_c (0.2 kPa) of the cilia-free substrate. The second relates to the chemical interactions between two solid surfaces[2], involving both hydrogen bonds and van der Waals forces in this case; the former concerns the interaction between the imide groups of PI and the hydroxyl groups of the cellulose in the rayon used here[3]. For a given material, surface treatment can also change the amounts of interactions. For example, after PI in the cilia region is treated with RIE, O_2 plasma enriches the oxygen content, which might increase the probability of hydrogen bonding; the relative ratio of the peaks O1s and C1s, changes from 0.5 to 1.7 (Supplementary Figure 11). The third relates to the actual contact area of cilia on the curvilinear woven surface, which can be determined by the thickness, surface roughness, length and density of

the cilia. Assuming that the ideal plane surface of a cilium wraps a cylinder with radius (R), the critical adhesion energy per unit area (γ_c), which is the minimum energy per unit area required for conformal wrapping, can be calculated (Supplementary Fig. S12 and S13)[4]. The γ_c becomes 0.28, 60, 280 and 950 mJ/m^2 for 300 nm, 1.8 μm, 3 μm and 4.5 μm thick cilia, respectively, which shows that thinner cilia result in more efficient wrapping behaviour and thereby larger contact area. When the surface of PI cilia is treated with O_2 plasma, it becomes rougher, which generally induces a smaller contact area[2]; the roughness factor changes from 1 nm to 67 nm after treatment for 7 min (Supplementary Figures 14 and 15). With the same thickness and surface roughness of cilia, which can be provided under the same RIE conditions for the surface treatment, longer and denser cilia induce more conformal contact area near the threads, as shown by the SEM images in Fig. 2a and 2b. The fourth relates to the residual stress of the samples generated during the fabrication process. When PI was treated with RIE and the supportive layer washed away, the free-standing film becomes concave in shape (Supplementary Figure S16), which is consistent with results previously reported in the literature using a PI cantilever tip[5], suggesting that the bending occurs by the applied compressive stress for the bottom surface and tensile stress for the top surface of the PI film in the fabrication process. This phenomenon lowers the value of γ_c. Friction during the detaching process also contributes to the increase in P_c because the detaching directions are not always normal to the cilia surfaces due to the turbulence of air-blowing and complex geometry of the cilia and the textile[6]. In the case of using PDMS glue, we believe the additional elastic layer near the cilia not only enhances the interfacial adhesion between the cilia and the textile but also protects the cilia mechanically, thereby enhancing the value of P_c; the larger amount of residual glue reinforces the cilia.

Supplementary Note 5: Surface analysis of PI film with and without O_2 plasma treatment

We investigated the surface of the PI film before and after O_2 plasma treatment by X-ray photoelectron spectroscopy (XPS). Fig. S11a shows the XPS survey scan spectra of the PI film with and without O_2 plasma treatment. The O_2 plasma increased the O1s/C1s peak ratio from 0.5 to 1.7, suggesting increased oxygen components. In the C1s XPS spectra, the peak at 286.3 eV attributed to C-O-C or C-OH increased sharply after O_2 plasma treatment (Supplementary Figures 11b and 11c). In particular, the peaks were much broader after O_2 plasma treatment, indicating increased full width at half maximum (FWHM) values. These results should be induced by the binding energy shift due to the effect of the adjacent structures and were also observed in other literature[7].

Supplementary Note 6: Calculated critical adhesion energy per unit area for conformal wrapping of cilia

We assume a thin film on a cylinder with a radius of R, as shown in Supplementary Figure 12a. For a thin film, the bending stiffness of homogeneous thin film was represented as

$$EI = \frac{1}{12} Ebh^3 \qquad \text{(Supplementary equation 1)}$$

where EI is the bending stiffness of a thin film, E is the elastic modulus of a thin film (PI ~2.5 Gpa), b is the width of a thin film and h is the thickness of a thin film. The bending stiffness (EI) increased sharply as a function of the thickness of the thin film, as shown in Supplementary Figure 10b. The adhesion energy per unit area (γ) for the conformal wrapping of a thin film is[4],

$$\gamma \geq \gamma_c = \frac{EI}{2R^2 b} \qquad \text{(Supplementary equation 2)}$$

where γ_c is the critical adhesion energy per unit area, which is the minimum energy required for the conformal wrapping of a thin film. At a fixed cilia width = 10 μm, γ_c was calculated for cases with different thicknesses of cilia and cylinder radii, and the values were compared to the experimental results (Supplementary Figures 5 and 12c). We estimated the γ required for the conformal wrapping of cilia on threads by fitting a line with the same γ_c values (red dotted line in Supplementary Figure 12c). In the case of cilia with thickness h = 0.3 and 1.8 μm at R = 100 μm, the conformal wrapping was established as shown in the SEM images of Supplementary Figure 5, and the corresponding γ_c value was approximately 60 mJ/m^2. For the conformal wrapping of cilia with thickness h = 3 and 4.5 μm at R = 100 μm, the estimated γ_c values were much higher, at 280 and 950 mJ/m^2, respectively.

We also assume a thin film on two overlapping cylinders with a radius of R to simplify the complex surface, as shown in Supplementary Figure 13a (left). In a cross-sectional scheme (Supplementary Figure 13a, right), the distance between the centres of two circles is 2d; the radius of a small circle generated by conformal contact with the thin film, is r_0, and the contact angle is θ. Then, the adhesion energy is[4]

$$\gamma \geq \gamma_c' = \frac{EI}{2R^2 b}\{1 + (1+\lambda)R^2/[(1-\lambda){r_0}^2]\} \qquad \text{(Supplementary equation 3)}$$

where

$$\lambda = r_0 d/\left\{(R + r_0)\sqrt{(R + r_0)^2 - d^2}\,\sin^{-1}[d/(R + r_0)]\right\} \qquad \text{(Supplementary equation 4)}$$

$$r_0 = \frac{d}{\sin\theta_0} - R \qquad \text{(Supplementary equation 5)}$$

When we transfer a thin film onto a complex substrate, the required adhesion energy is much higher than in the case shown in Supplementary Figure 12, as the rough morphology limits the conformal contact, requiring an extremely small bending radius of the thin film. Supplementary Figures 13b and 13c show two cases for cylinders with R = 150 or 10 μm.

The results show that a rough surface (with smaller R) requires much higher adhesion energy for the conformal wrapping of thin film. Therefore, we introduced glue for conformal contact with the complex surfaces to enhance the adhesion energy, as shown in Figure 3. The glue enabled the attachment of the device on a cotton swab and a stone through its synergistic effect with the cilia.

Supplementary Note 7: Undesirable deformation of PI film by residual stress

Undesirable deformation of the patterned substrate transferred onto a textile right side up was observed at the edge of the cilia or PI film, as shown in Supplementary Figure 16. When we consider the applied stress in a thin film released from the substrate, the total intrinsic stress is the summation of a constant mean stress (σ_0) and a gradient stress (σ_1). Under a linear stress profile in a thin film, the total intrinsic stress distribution σ (z) is[5]

$$\sigma\,(\mathrm{z}) = \sigma_0 + \sigma_1 \frac{z}{t/2} \qquad \text{(Supplementary equation 6)}$$

where z ∈ (-t/2, t/2) is the coordinate normal to the surface of thin film with the origin at the mid plane of the thin film, and t is the thickness of the thin film. In our study, the stress gradient in the PI film (cilia and patterned substrate) by film formation and various fabrication processes including O_2 plasma treatment should generate compressive stress on the bottom surface and tensile stress on the top surface, inducing upward bending of the PI thin film after dissolving the supportive layer and drying the solvent. Then, the latter term in Supplementary equation 6 becomes positive, indicating residual stress with a positive stress gradient.

Supplementary Note 8: Numerical mechanical modelling

To investigate the damping behavior of the peripheral cilia on the structure of serpentine electrode with textile under mechanical deformation, FEM analysis was performed using a commercial ANSYS program. FEM modeling was constructed on the base of the SEM images, which is illustrated in Supplementary Figures 23 and 24. The textile has plain weave shape and is modeled as the mesoscopic scale type. The approximate shape, size and spacing of the thread were obtained by analyzing the SEM images. Based on the tensile direction of textile, the specific area of the electrodes, which has horizontal and vertical orientation, was modeled. Anisotropic properties of thread was derived from an ideal thread model of continuous fibers and calculated from the thread constitutive equation[8]. Material properties data used in the study are described in Supplementary Table 2. All materials except thread were regarded as elastic and isotropic materials. A frictionless contact was defined for thread-thread, thread-electrode and thread-cilia, and the attached area of cilia on thread was assumed for perfect bonding state. The left edge of the textile was constrained for the x, y, and z axis, and the right edge of the textile was constrained for the y and z axis respectively. The load displacement of the x-axis direction was applied on the right edge of the textile with a 9% tensile strain. The structure was modeled with the SOLID185 element which has 8 nodes, and it was assumed that there is no deformation by the residual stress.

Supplementary Note 9: Heat dissipation test

Supplementary Figures 28a and 28b show the temperature distribution of the electrode at DC 15 V with no strain and with tensile strain ε = 15.3%. The maximum temperature for the stretched electrode (~50 °C) was higher than for the non-stretched electrode (~45 °C). We believe that the increased resistance caused by tensile strain, as observed in the results shown in Fig. 5, induces the higher maximum temperature.

Heat dissipation is an issue in flexible electronics[9]; it is desirable to use a minimal amount of polymer with comparably low thermal conductivity (κ) and high specific heat capacity (c) to allow efficient air-cooling. We investigated the cooling behaviour of the encapsulated electrode on a textile with PDMS glue ($PDMS_g$) and PDMS film ($PDMS_f$) with the application of air blowing by fans at different speeds. The maximum temperature distribution was observed upon applying DC 15V, followed by removing the voltage source, as shown in Supplementary Figures 28c and 28d. From the cooling curves, the time constant values, τ_1 and τ_2, and thermal decay time, t, required to reach 80% at maximum temperature (48-51 °C) for the samples were extracted as shown in Supplementary Table 4. In a lumped heat capacity system, assuming that the temperature of the whole body is constant at any point and changes uniformly with time, the time constant, τ, is[10]

$$\tau = \frac{\rho \cdot c_p \cdot V}{h \cdot A} \qquad \text{(Supplementary equation 7)}$$

where ρ is the density, c_p is the specific heat capacity, V is the volume of the metal body, A is the surface area of the body, and h is the heat transfer coefficient between the body and medium. According to Supplementary equation 7, as τ is proportional to the mass ($m = \rho \cdot V$) and c_p, using a small amount of PDMS with low thermal conductivity (κ) and high c_p should induce faster thermal decay (shorter τ). The κ and c_p values for the materials are shown in

Supplementary Table 5. Using $PDMS_f$ and $PDMS_g$ results in different cooling behaviour fitted by first-order and second-order exponential functions, respectively. The thermal loss between the heated objects and their surroundings is generally described as a combined effect of three fundamental mechanisms (conduction, convection and radiation) and expressed by a simple exponential function (Newton's law of cooling). Using $PDMS_g$ showed the deviation from Newton's law of cooling at relatively high temperature, which required a second-order exponential function for exact fitting with the experimental data, in contrast to using $PDMS_f$. In our system, a different medium (air or PDMS) could induce changes in the thermal decay process.

Supplementary Note 10: Basic electrical properties of IGZO-based devices

Our device shows typical transfer characteristics of n-type TFT and a voltage transfer curve of the inverter consisting of enhancement-type load and drive transistors. The field effect mobility and threshold voltage of the TFT at V_{DD} = 1 V were 4.6 $cm^2V^{-1}s^{-1}$ and 1.5 V, respectively.

Supplementary references

1 http://www.mit.edu/~6.777/matprops/matprops.htm for PI, PDMS, Cr and Au. http://www.engineeringtoolbox.com/specific-heat-capacity-d_391.html for cellulose.

2. Bhushan, B., Adhesion and stiction: Mechanisms, measurement techniques, and methods for reduction. *J. Vac. Sci. Technol. B* **21,** 2262-2296 (2003).

3 Gardner, D. J., Oporto, G. S., Mills, R., & Samir, M. A. S. A., Adhesion and Surface Issues in Cellulose and Nanocellulose. *J. Adhesion Sci. Technol.* **22**, 545-567 (2008).

4 Kim, D,-H. *et al.* Dissolvable films of silk fibroin for ultrathin conformal bio-integrated electronics. *Nature Mater.* **9,** 511-517 (2010).

5 Thuau, D., Koutsos, V., and Cheung, R., Stress relaxation of polyimide (PI) cantilevers using low energy ion bombardment. *Soft Mater*. **11,** 414-420 (2013).

6 Tian, Y. *et al*. Adhesion and friction in gecko toe attachment and detachment. *Proc. Natl Acad. Sci. USA* **103,** 19320-19325 (2006).

7 Ektessabi, A. M., Hakamata. S., XPS study of ion beam modified polyimide films. *Thin Solid Films* **377-378**, 621-625 (2000).

8 Ning, P. and David, B., Physical properties of twisted structures. II. Industrial yarns, cords, and ropes. *J. Appl. Polym. Sci.* **83,** 610-630 (2002).

9 Moore, A. L., and Shi, L., Emerging challenges and materials for thermal management of electronics. *Mater. Today* **17,** 163-174 (2014).

10 Perumal, R. W., Nithiarasu, P., Seetharamu, K., *Fundamentals of the finite element method for heat and fluid flow* Ch. 6 (John Wiley & Sons, Ltd., Chichester, 2004).
